# Nutraceutical Concepts and Dextrin-Based Delivery Systems

**DOI:** 10.3390/ijms23084102

**Published:** 2022-04-07

**Authors:** Gjylije Hoti, Adrián Matencio, Alberto Rubin Pedrazzo, Claudio Cecone, Silvia Lucia Appleton, Yousef Khazaei Monfared, Fabrizio Caldera, Francesco Trotta

**Affiliations:** Department of Chemistry, University of Torino, Via P. Giuria 7, 10125 Torino, Italy; gjylije.hoti@unito.it (G.H.); adrian.matencioduran@unito.it (A.M.); alberto.rubinpedrazzo@unito.it (A.R.P.); claudio.cecone@unito.it (C.C.); silvialucia.appleton@unito.it (S.L.A.); yousef.khazaeimonfared@unito.it (Y.K.M.); fabrizio.caldera@unito.it (F.C.)

**Keywords:** disease, nutraceuticals, nano-carrier, starch, linear dextrin, cyclic dextrin, nanosponges, drug delivery, nutraceutical delivery

## Abstract

Nutraceuticals are bioactive or chemical compounds acclaimed for their valuable biological activities and health-promoting effects. The global community is faced with many health concerns such as cancers, cardiovascular and neurodegenerative diseases, diabetes, arthritis, osteoporosis, etc. The effect of nutraceuticals is similar to pharmaceuticals, even though the term nutraceutical has no regulatory definition. The usage of nutraceuticals, to prevent and treat the aforementioned diseases, is limited by several features such as poor water solubility, low bioavailability, low stability, low permeability, low efficacy, etc. These downsides can be overcome by the application of the field of nanotechnology manipulating the properties and structures of materials at the nanometer scale. In this review, the linear and cyclic dextrin, formed during the enzymatic degradation of starch, are highlighted as highly promising nanomaterials- based drug delivery systems. The modified cyclic dextrin, cyclodextrin (CD)-based nanosponges (NSs), are well-known delivery systems of several nutraceuticals such as quercetin, curcumin, resveratrol, thyme essential oil, melatonin, and appear as a more advanced drug delivery system than modified linear dextrin. CD-based NSs prolong and control the nutraceuticals release, and display higher biocompatibility, stability, and solubility of poorly water-soluble nutraceuticals than the CD-inclusion complexes, or uncomplexed nutraceuticals. In addition, the well-explored CD-based NSs pathways, as drug delivery systems, are described. Although important progress is made in drug delivery, all the findings will serve as a source for the use of CD-based nanosystems for nutraceutical delivery. To sum up, our review introduces the extensive literature about the nutraceutical concepts, synthesis, characterization, and applications of the CD-based nano delivery systems that will further contribute to the nutraceutical delivery with more potent nanosystems based on linear dextrins.

## 1. Introduction

Industrialization and changing work cultures have caused numerous air and water pollutions, soil, and food contamination because of the extensive use of various harmful man-made items such as chemicals, heavy metals, electromagnetic waves, etc. At the same time, economic development has simultaneously drastically changed human lifestyles, which have become fast-eating cultures with decreasing nutrient quality. Therefore, due to nutritional deficiencies, there is an increase in the development of several diseases such as diabetes, obesity, various cancers, neurodegenerative diseases, heart disease, physiological problems, hypertension and dyslipidemia, chronic and vascular diseases, osteoporosis, arthritis, and many others.

Based on the fact that the raised demands for health care have dramatically increased, more and more people eat minimally processed foods such as vegetables, fruits, and other plant foods, taking dietary supplements or nutraceuticals instead [1,2,3,4,5,6]. The maintenance of the normal functioning of human body, recognized as a global issue, is reached by obtaining appropriate nutrients from various foods [1,6,7].

For centuries, the major concern of humankind around the world has been the research, development, and commercialization of nutraceuticals, functional food ingredients, and dietary supplements. There are a few challenges to defining the health benefits of certain foods, improving immune function, preventing specific diseases, and reducing side effects and health care costs [3,5,8,9]. The estimation of the mechanism of action and the efficacy of nutraceuticals have been encouraged as a consequence of the challenges of nutraceuticals with safety and health claim trials. Nutraceuticals comprise prebiotics, probiotics, polyunsaturated fatty acids, antioxidants, herbal products, etc. [10,11]. As more consumers use nutraceuticals for disease prevention [12], their efficacy as therapeutic agents is determined by different pathways. Based on drug studies and knowledge, the requirements to achieve the therapeutic purpose comprise the improvement of bioavailability, biocompatibility, solubility, loading efficacy, and toxicity as well as controlling the release, broadening the activity, adjusting the pharmacokinetics (PKs) of the drugs, etc. [13]. Therefore, the therapeutic efficacy of a drug can be improved, and toxic effects can be reduced by developing a drug delivery system. The drug delivery system can be controlled and targeted. Among various drug delivery systems, the molecular nano-carrier has produced great interest within the scientific world [14]. This review will explicitly focus on the application of dextrins in the drug delivery industry because of their non-toxic, biodegradable, and biocompatible nature, water solubility, or high encapsulation for swelling caused by simple chemical modifications [15,16]. These findings on well-explored drug delivery systems will enable the successful development of nutraceutical delivery systems, which are lacking due to the existing contradictory information regarding the nutraceutical term [17].

Dextrins, starch derivatives [18], are well-known for their great potential to develop hydrogels because of their efficient absorption related to degradation by amylases, and their proven clinical tolerability [19]. According to the molecular structure, dextrin can be divided into linear, branched dextrin, and cyclodextrin [20]. Dextrins consist of D-glucose units linked primarily by α (1,4)-glycosidic bonds [21], and branched segments linked by α (1,6)-glycosidic bonds [22]. Cyclodextrins (CDs) and linear dextrins have the same physicochemical, and biological characteristics, but CDs due to their cyclic structure are more resistant to non-enzymatic hydrolysis [23]. CDs are characterized by a typical toroidal cone shape with a lipophilic interior and hydrophilic exterior. Therefore, this peculiar structure enables CDs to form inclusion complexes with the compounds that have the size and polarity compatible with CDs structure [24]. Starch hydrolyzates, with the values of dextrose equivalent (DE, 1–20), are known as maltodextrins. Maltodextrin is a linear dextrin consisting of linear (amylase) and branched (amylopectin) carbohydrates [22,25,26,27]. Whether the linear dextrin acts comparably as CDs, with one side of the molecule being hydrophilic and the other hydrophobic, is a question that has been addressed over recent decades. A study suggested that the dextrin chains are amphiphilic ribbons, and under certain conditions, the hydrophobic surfaces are noticed by either hydrophobic or amphiphilic molecules [28]. However, viscosity drop during storage, poor solubility, uncontrolled hydration rate, and microbial contamination are some limitations of dextrin [29]. One approach that was applied to address the limitations issue is the use of chemical modification [30,31]. Chemical modification of dextrin can lead to the formation of dextrin polymers and nanosponges (NSs). Nanosponges (NSs) are hyper-cross-linked polymers that can be obtained by reacting CDs or maltodextrins with an appropriate cross-linking agent [32].

As explored from the historical evolution of NSs, cyclodextrin (CD) polymers have found their applications as food component carriers in the 1990s. Whereas CD-NSs are widely explored as drug delivery systems in the new millennium [33]. CD-NSs have shown more advantages compared to the native CDs in entrapping guest molecules, reducing their side effects, improving their stability, extending their release, etc. [34]. This is because CD-NSs, as chemically three-dimensional nanoporous polymeric networks, have various attractive features for use as hydrogels [35]. This strategy facilitates the slower drug elution and maintains a high concentration of drugs in the surrounding tissues over an extended period [36,37]. The CD: cross-linking agent molar ratio affects the nanochannels produced, the swelling, and therefore the loading capacity, and the drug release [35]. The diffusion process in the cross-linked polymer or dense macromolecular networks is slow, and thus the drug release is prolonged and controlled [38]. A free hydrophilic drug, that can freely spread in the aqueous medium, interacts with the hydrophilic zone of the biological membrane model but is unable to pass through the lipophilic layer of the same membrane. For instance, in the case of ester-bridged NSs based on β-CD, the electrostatic interactions of the carboxylic groups of dianhydride bridges with polar moieties of hydrophilic drugs can provide interaction with the hydrophilic layer of the membrane, while the inclusion complex formation with the lipophilic part can provide close interaction with the hydrophobic layer of the membrane, providing a high permeation of the drug [14,35]. Due to electrostatic repulsion, the polymer swells, and the volume of its network increases. Consequently, this increases the pore size of the NSs, and the drug is released [39]. CD-NSs have evolved alongside generations, from the plain NSs to modified NSs, to stimuli-responsive CD polymers, and to molecularly imprinted CD (MIPs-CD) polymers. Delivery challenges for each native CD have been addressed through the CD-NSs generations which have improved the delivery kinetics for most of the therapeutic agents. CD-NSs are well-known delivery systems of several nutraceuticals such as quercetin [40], curcumin [41], resveratrol [42], thyme essential oil [43], melatonin [44], etc.

The potential of any innovative or discovery process is greater when the obstacles between two of its basic ingredients, such as having an idea and testing it, are fewer [45]. Thus, our idea to review the nutraceuticals concept, the CD-NSs for their delivery, along with this entry, will support the use of maltodextrins modification for nutraceuticals delivery. This review attempts to summarize the recent headway on this new health care concept. As Hulda Regehr Clark quoted in her book, “The Cure for All Advanced Cancers”: “*…never take defeat. When all is lost, try something new. Life is too precious to let it slip away from lack of initiative or plain inertia*”.

## 2. Nutraceuticals

### 2.1. What Are Nutraceuticals?

**Figure 1:**
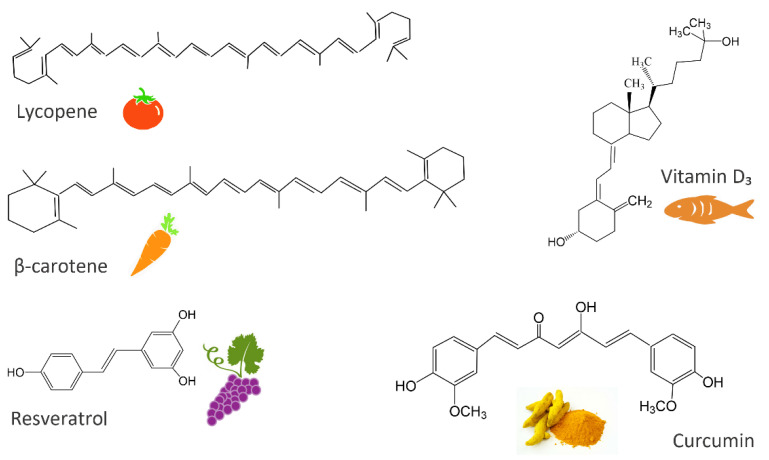
Nutraceuticals.

Nutraceuticals are natural bioactive or chemical compounds that possess valuable biological activities and demonstrated physiological benefits. Therefore, they offer promotion of the body’s natural healing, prevention, and treatment of the disease [5,46,47]. The introduction of many nutraceuticals, as presented in Figure 1, has undoubtedly caused an increasing challenge for nutritionists, physicians, food technologists, and chemists [46] based on the goal of providing a positive impact on human health [48].

The fascinating topic of the food supply has existed throughout history [46]. The connection between the use of relevant foods and health was established by the father of modern medicine, Hippocrates (460–377 BC), more than 2500 years ago, who made the statement “*Let food be thy medicine and medicine be thy food*” [1,3,5]. The term “Nutraceutical” originates from two broad terms such as “Nutrition” and “Pharmaceutical” [4,6,9,17,49,50,51,52], and was coined in 1989 by Stephen DeFelice, MD, founder, and chairman of the Foundation for Innovation in Medicine (FIM), Cranford, New Jersey. According to him, the term nutraceutical is defined as “*a food or a part of a food that provides medical or health benefits, including the prevention and the treatment of a disease*”, because in his opinion “*the nutraceutical revolution will lead us into a new era of medicine and health, in which the food industry by the year 2000 will become a research-oriented one similar to the pharmaceutical industry*” [45]. This concept has been presented as a modern approach to food science. However, the definition of nutraceuticals and a legitimate assessment of their potential in medicine are still in opposition [17]. 

**Figure 2:**
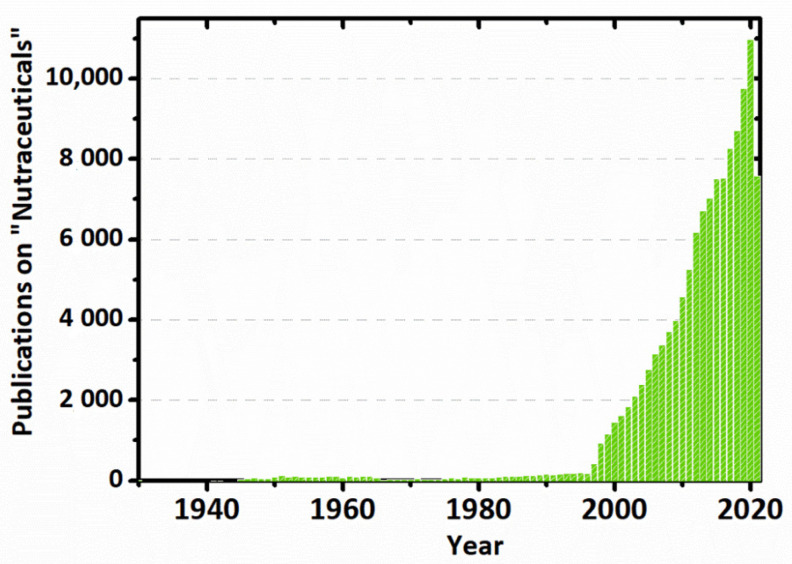
Graph representing the number of research papers (found in PubMed) published on nutraceuticals per year.

Stephen L. DeFelice, in a 2014 lecture, admitted that the clinical trials, which have proven the malfunctioning of the dietary supplements and diets, may not have been designed properly. From his standpoint, the reason why dietary supplements and diets do not work is that the cell is not deficient in them and does not need them. Further, he revealed his theory called, “*the cell-nutraceutical acceptance-rejection theory*” related to the lack of efficacy and toxicity. It is a self-explanatory theory, probably explained by the fact that any medication is unlikely to produce benefits if it does not cause harm. In the end, DeFelice stated that the nutrition area will be ongoing, and there is just a need for some new creative thinkers [53,54]. The research in the nutraceuticals area, judging from the number of journal articles indexed in PubMed, has increased steeply since 2000 and is continuously developed [17,53], as presented in Figure 2.

### 2.2. Nutraceuticals vs. Other Definitions/Regulations

**Figure 3:**
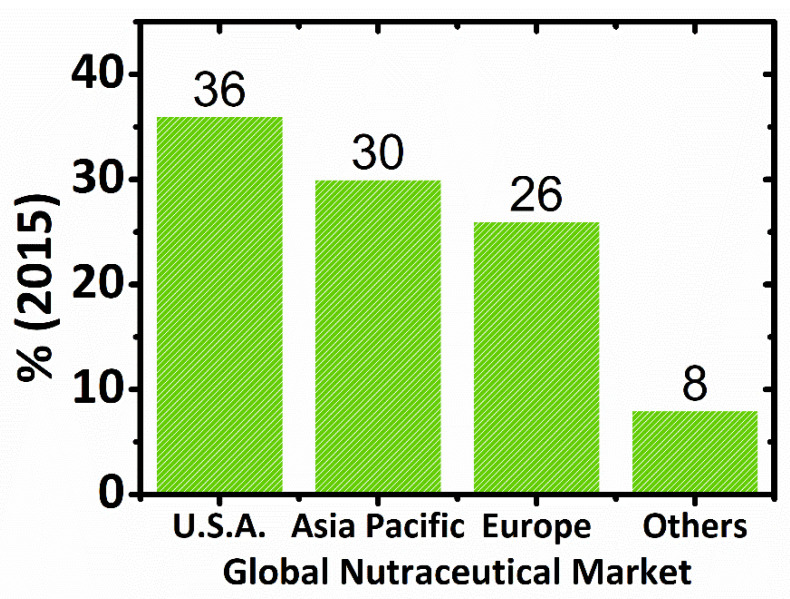
Global Nutraceutical Market by Region (%) in 2015: U.S.A. (36%), Asia Pacific (30%), Europe (26%), and others include the rest of the world (8%) [83].

The term nutraceutical has no regulatory definition, and the existing contradictory information is generating confusion about the possible effective use of these products. This may be due to a lack of studies on possible mechanisms of action and in vivo research confirming the declared beneficial health effects on specific pathological conditions, as mentioned in the lecture of DeFelice in 2014 [17,49,51]. Therefore, this situation has encouraged the utmost interest for the need of assessing the safety, mechanism of action, and efficacy of nutraceuticals with clinical data. There has been a lot of confusion between the term “nutraceuticals” and the others such as “functional foods”, dietary supplements”, “designer foods”, “medical foods”, “pharmafoods”, “phytochemicals” etc. (Figure 3). Nutraceuticals, standing between pharmaceuticals and foods, have experienced challenges with safety and health claim trials [11,55]. In comparison to the pharmaceuticals, uni-targeted pure compounds with high-dose use, nutraceuticals are multi-targeted mixtures existing at low concentrations [47]. While the concepts of nutraceuticals, medical or functional foods, and dietary supplements do not have a clear accepted definition, they can most often be used in an exchangeable way [5]. Certain organizations proposed several definitions for nutraceuticals as one of the most active areas of research with a deficiency of a favorable regulatory environment. The understanding of the modern concept of functional food related to the maintenance of health in the general population was proposed by the Japanese academic society in the early 1980s, which implemented the legislation “Foods for Specified Health Use (FOSHU)” [56]. 

The definition for “functional food” describes, “*food products fortified with special constituents that possess advantageous physiological effects*”, whereas, “*the approved health claim; recommended daily intake of the food; nutrition information; guidance on healthy eating; a warning against excessive intake, if necessary; any other special precautions relating to intake, preparation or storage; and other information*”, has been the completed FOSHU label. The other legislations that have influenced the dissemination of information to consumers about the relationship between the diet and health in food regulations are the Functional Food Science in Europe (FUFOSE) Concerted Action, NLEA in 1990 [57], the Dietary Supplement Health and Education Act (DSHEA) in 1994 [58], FDA Modernization Act, in 1997 [59], and Functional Food Center (FFC) [56,60,61,62,63,64,65,66,67,68]. 

Functional foods provide the required amounts of vitamins, fats, proteins, carbohydrates, etc., that the human body needs for healthy survival. The functional foods are called a Nutraceutical when they assist in the precaution and the treatment of any disease or disorder [69]. As dietary supplements are not considered to treat, cure, or prevent disease [66], the definition of nutraceuticals as, “*those diet supplements that deliver a concentrated form of a presumed bioactive agent from a food, presented in a non-food matrix, and used to enhance health in dosage that exceeds those that could be obtained from normal food*” is used to distinguish whole foods from the isolated components derived from them and to create the category of nutraceuticals for dietary supplements that can obtain pharmacological effects [65,70].

Medical foods are a specific category of therapeutic agents that are intended for the specific dietary management of the disease [66,69]. The term “phytochemicals” refers to a group of plant secondary metabolites that may account for numerous beneficial health effects [71,72] and have the potential of being incorporated into foods, nutraceuticals, or pharmaceuticals [73]. The United Kingdom, Germany, and France are the first countries considering that diet is a more important factor than exercise in achieving good health.

The health ministry of Canada, the Ministry of Agriculture, Fisheries and Food of Britain, the Merriam Webster Dictionary, the dietary supplement industry, the North American Veterinary Nutraceutical Council Inc. presented various definitions that modified the meaning of the term “nutraceutical” [2,53,74,75]. 

“*A substance that is cultivated/produced/extracted or synthesized under optimal and reproducible conditions and when administered orally to patients, would provide the nutrient required for bringing altered body structure and function back to normal, thus improving the health and wellbeing of the patients*”, is a more appropriate definition for nutraceuticals based on the abovementioned definitions [9].

Nutraceuticals have the advantage over foods and drugs because they may comprise more than a single food or plant component, that may be a contributing active ingredient, but their regulation varies widely around the world [2,9,19]. Nutraceuticals comprised herbal products, isolated nutrients, dietary supplements, diets, genetically engineered foods and processed products such as soups, cereals, and beverages [46] but then, with the passage of the *Dietary Supplement Health* and *Education Act of 1994,* was expanded to include minerals, herbs, vitamins, and other botanicals, aminoacids and any dietary substance [76]. As evidenced by the number of existing nutritional terms that are mentioned above and published elsewhere in the literature, the presence of a consistent definition, to what “functional foods”, “nutraceuticals”, and other terms mean is essential to properly educate the public about these products which are adequate to deal with future chronic disease prevention and care [56].

### 2.3. Global Market of Nutraceuticals

Even though there is a disagreement among experts as to what a nutraceutical is, the movement regarding nutraceuticals in the global market is “unstoppable” [77]. The lack of strict regulations controlling nutraceuticals is the main factor that leads to inflating the market share of these products [78]. The beneficial role of nutraceuticals and healthy foods in wellness promotion and disease prevention has been demonstrated by nutritionists and health professionals and has led to an increase in the number of nutraceuticals worldwide [79]. Consumers, thanks to the information available on health-related foods and supplements, spend billions of dollars each year purchasing them. It is significant to evaluate the global nutraceutical market that engenders constant controversy [73]. The industry of nutraceuticals is broken down into functional foods, natural or herbal products, and dietary supplements.

An USD 80 billion nutraceuticals market was identified by the Nutrition Business Journal (NBJ) in 1995 and as USD 91.7 billion in 1997. In 1996, more than USD 6.5 billion were invested in dietary supplements by U.S. consumers, almost doubling this market to USD 12 billion in 1998. Dietary supplements (19.5% per year) and natural or herbal products (11.6% per year) were the most rapidly growing segments of the industry. According to BCC Research, in 2007, the global nutraceuticals market was worth USD 117.3 billion, whereas, in 2013, it was USD 176.7 billion. Nutraceutical foods were the largest market segment in 2007, worth USD 39.9 billion [2,50,70,75]. In 2004, the global nutraceuticals market was estimated to be approximately $106 billion [80], USD 142.1 billion in 2011 [9], and USD 117 billion in 2017 [78]. The global market for food supplements was estimated to be worth between EUR 45 billion and EUR 50 billion in 2009, whereas the EU market was between EUR 8.2 billion and EUR 8.6 billion [81]. Emerging nutraceuticals technology has created a global market with impressive growth rates, with the United States followed by Japan and the European Union as major markets. In 2016, the global nutraceutical industry has experienced an increment of USD 198.7 billion [78] and, in 2018, USD 231 billion is projected to grow at a compound annual growth rate (CAGR) of 7.8% from 2018 to 2023 [82]. Figure 3 presents the global nutraceutical market by region (%) in 2015.

### 2.4. Classification of Nutraceuticals

**Figure 4:**
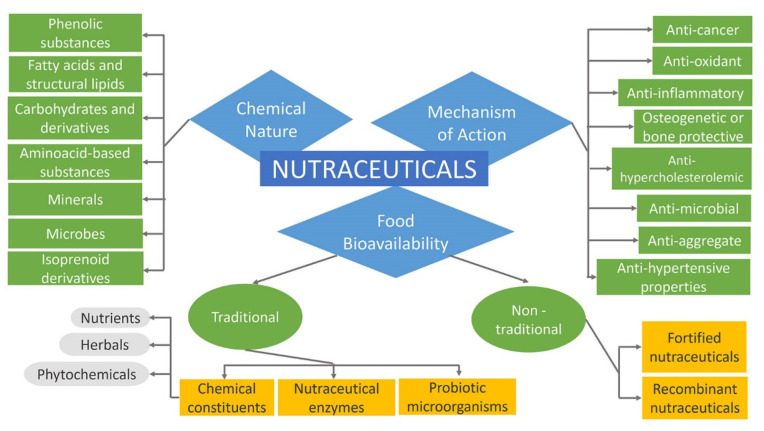
Classification of Nutraceuticals.

Nutraceuticals can be classified into broad classes based on food bioavailability, mechanism of action of active component, and chemical nature, as presented in Figure 4. Further, they are classified into several sub-classes as follows [84,85]. More comprehensively, nutraceuticals, regarding their promise, can be classified in two ways such as potential nutraceuticals including the majority of nutraceuticals that maintain a promise of a particular health or medical benefit, and established nutraceuticals related to the attainment of the efficient clinical data that prove these benefits [2,6,86]. For instance, folic acid was first considered a potential nutraceutical. Subsequently, it was altered to an established nutraceutical after the release of sufficient clinical evidence that confirmed neural tube defects prevention [45].

#### 2.4.1. Nutraceuticals Based on Food Bioavailability

Regarding the food source, nutraceuticals can be divided into traditional and non-traditional.

Traditional nutraceuticals comprise food that is sourced directly from nature without any further modification. This group, for example, many fruits, fish, grains, tomatoes, salmon or soy, tea, chocolate, which contain various constituents such as fatty acids, lycopene, omega-3, saponins, etc., deliver benefits beyond basic nutrition. Chemical constituents, probiotic microorganisms, and nutraceutical enzymes are several types of traditional nutraceuticals. Nutraceutical enzymes are proteinous in nature, specific in action, and are produced by cells of the body. They can also increase the rate of metabolic activity occurring inside the cells [54,87]. The symptoms of medical conditions such as hypoglycemia, blood sugar disorders, digestive problems, and obesity can be eliminated by adding enzyme supplements to the diet. These enzymes are derived from animal, plant, and microbial sources [88]. Probiotic refers to viable microorganisms that have a vital position in the medical field by making the gastrointestinal tract (GT) more favorable to processes such as metabolism and absorption. Probiotics are counted as an impressive number of microbial species that eradicate toxic flora inside the intestine because of their tolerance to acid and bile salts. With respect to foods, probiotics are considered as, “*viable preparations in foods or dietary supplements to improve the health of humans and animals*” [54,89,90]. In addition, chemical constituents incorporate nutrients, herbals, and phytochemicals. Nutrients are substances with established nutritional functions to sustain the life or health of a person, animal, or part of the body. These substances are antioxidants, minerals, vitamins, amino acids, and fatty acids. Herbals are herbs or botanical products subjected to treatments such as distillation, extraction, fractionation, purification, concentration, etc. They can be found in berries, leaves, roots, and flowers as various parts of plants that are used for medicinal purposes. The combination of herbal products with nutraceuticals can treat many chronic disorders [52,53,54,84]. Phytochemicals have become more popular as a result of the increment of studies on nutrients. Based on a wide variety of chemical compounds that plants carry, phytochemicals include phenolics, nitrogen-containing compounds, alkaloids, and terpenoids. When phytochemicals are present in plant-rich diets, they lower morbidity and mortality in adult life [71,91].

Further, agricultural or food engineering and product development are the main factors of the appearance of non-traditional nutraceuticals on the market. They are foods enriched with supplements or biotechnologically designed crops to raise the nutrients and ingredients, comprising orange juice fortified with calcium, cereals with added vitamins or minerals, β-carotene-enriched rice, soybeans, and flour with added folic acid [76,92]. They are arranged into fortified and recombinant nutraceuticals. Fortified nutraceuticals comprise fortified foodstuff from agricultural production or the addition of the compatible nutrients to the main ingredients such as flour fortified with calcium, minerals added to cereals, milk fortified with cholecalciferol to treat deficiency of vitamin D, etc. Apart from these, recombinant nutraceuticals involve foodstuffs which are a source of energy. These nutraceuticals, produced using various biotechnological processes, comprise cheese, bread yogurt, vinegar, fermented starch, etc. The cheese and bread, through a fermentation process, extract the enzyme useful for providing necessary nutrients at an optimum level [54,88,93].

#### 2.4.2. Nutraceuticals Based on Chemical Nature

These types are classified based upon nutraceutical chemical nature, more specifically upon functional groups. Based on their primary and secondary metabolite sources, there include several large groups such as isoprenoid derivatives, phenolic substances, fatty acids, structural lipids, carbohydrate derivatives, amino acid derivatives, microbes, and minerals, which provide a basis for subclassification [71,84,94]. Justus von Liebig, the German chemist, proposed that the nutritive value of food and feed can be predicted from the knowledge of the chemical composition of energy-yielding substances such as carbohydrates, fats, proteins, and a few minerals. These substances represent the essentials of a nutritionally adequate diet. The basic structure of proteins, carbohydrates, lipids, vitamins is made up largely of six elements such as hydrogen, oxygen, carbon, nitrogen, phosphorus, and sulfur. The atoms of the aforementioned elements, in organic molecules, are held together by covalent bonds. These bonds are formed when two atoms share a pair of outer orbital electrons and each covalent bond allows the organic molecule to serve as the energy source of the body [95].

##### Isoprenoid Derivatives

Isoprenoids, also known as terpenoids, are synthesized from a universal compound isopentenyl diphosphate (IPP) and belong to a vast group of secondary metabolites such as carotenoids, polyprenyl alcohols, sterols, ubiquinone (coenzyme Q), prenylated proteins, and heme A [96]. The evidence that isoprenoids are extremely diverse in chemical structure is demonstrated by the characterization of over 23,000 individual isoprenoid compounds and the announcement of hundreds of new structures each year [97].

##### Phenolic Substances

Phenolic compounds or polyphenols, referring to more than 8000 compounds found in the plant kingdom, are plant secondary metabolites that possess at least an aromatic ring with one or more hydroxyl functional (-OH) groups. They are essential for the growth, development, and reproduction of plants. Their classification can be based on the source of origin, biological function, and chemical structure. To render comprehensible, the classification according to their chemical structure is taken into account. Polyphenols can be divided into several sub-groups such as simple phenols and phenolic acids (hydroxybenzoic and hydroxycinnamic acids), flavonoids (flavones, flavonols, flavanones, isoflavones, flavanonols, anthocyanidins, tannins), stilbenes (resveratrol), and lignans found in plants and foods of plant origin [98,99,100]. Rich sources of phenolic compounds are fruits, vegetables, whole grains, tea, chocolate, wine, herbs, spices, cereals, oils, seeds, legumes, and others [101,102,103]. As there are 100 glucosinolates, 200 phytoestrogens, 700 carotenoids, and 4000 mono- and polyphenolics, it is impossible to cover all the information about their mode of action and clinical activity [104].

##### Fatty Acids and Structural Lipids

Lipids are a heterogeneous group of molecules that are insoluble in water but soluble in organic solvents. They are structurally quite diverse, ranging from simple short hydrocarbon chains to more complex molecules. Their classification includes eight categories such as fatty acyls, glycerophospholipids, sphingolipids, glycerolipids, saccharolipids, sterol lipids, prenol lipids, and polyketides [105,106,107]. One of the most fundamental categories of biological lipids is the fatty acyl structure representing the major lipid building block of complex lipids [108]. The category of fatty acyls contains fatty acids, aldehydes, alcohols, esters, and amines [107]. Fatty acids are the main constituents of the human cell [109]. They are hydrocarbon chains of varying lengths and degrees of unsaturation, with a methyl group (-CH_3_) at one end and a carboxyl group (-COOH) at the other end. The α carbon is the carbon atom next to the carboxyl group, whereas the β carbon is the subsequent one. The last position of fatty acids (-CH_3_ group) is designated as omega (ω) carbon. The first step in the synthesis of fatty acids involves the conversion of acetyl-CoA to malonyl-CoA by the enzyme acetyl-CoA carboxylase. Fatty acids can be classified, according to the presence or absence of double bonds, as saturated without double bonds (the most common ones contain 12 and 22 carbon atoms), monounsaturated fatty acids with one C = C located in different positions, and polyunsaturated fatty acids (PUFAa) with more than one double bond. The unsaturated fatty acids can be classified based on the configuration of the double bonds as cis or trans. Further, they can be categorized, based on the first double bond position from the fatty acid methyl-end, as ω-3 PUFAs including primarily α-linolenic acid (ALA) and its metabolic products such as eicosapentaenoic acid (EPA) and docosahexaenoic acid (DHA), as ω-6 PUFAs including primarily linoleic acid (LA) and its derivative arachidonic acid (AA), and as ω-9 monounsaturated fatty acids including primarily oleic acid. These are considered major fatty acids among others. PUFAs, with the first double bond on C3 (α-linolenic acid) and C6 (linoleic acid) from the methyl end, are intended essential because the human body cannot synthesize them, therefore, they have to be taken from a diet [110,111,112]. The predominant PUFAs, in all diets, are the ω-6 fatty acids with the linoleic acid as their representative whereas α-linolenic fatty acid is the precursor of other ω-3 long-chain PUFAs [109]. Vegetable oils, dairy products, meat products, eggs, soybean, certain seaweeds, grains, and fatty fish or fish oils are the most important dietary sources of fatty acids [110,113].

##### Carbohydrate Derivatives

Carbohydrates, derived from plant sources, are the most abundant class of organic compounds found in living organisms. They are divided into sugars with a degree of polymerization (DP) 1–2, oligosaccharides with a DP 3–9, and polysaccharides with a DP ≥ 10. Sugars include monosaccharides, disaccharides, and sugar alcohols, whereas oligosaccharides include α-glucans and non-α-glucan. As for polysaccharides, they are classified into starch (α-1:4 and 1:6 glucans), and non-starch, or the cell-wall polysaccharides (NSPs). The storage carbohydrates (starch, oligosaccharides and sugars), and the cell-wall polysaccharides (derived from plants, fungi and algae) are two important classes of plant carbohydrates with a contrasting but an important impact on human health [114,115,116]. The major storage carbohydrate in plants is starch. Starch is a mixture of two glucose polymers such as unbranched amylose comprising (1→4) α-linked chains of up to several thousand glucose units and highly branched amylopectin comprising (1→4) and (1→6) α-linkages of over 100,000 glucose residues. On the other side, cell-wall polysaccharides may be widely grouped into three major categories such as hemicelluloses, cellulose, and pectic polysaccharides. They are mainly found in the plant cell wall and consist of certain monosaccharides residues joined to each other by glycosidic linkages. Plant cell walls are highly complex structures that determine the quality characteristics of many plant-based foods. As one of the main plant cell wall components, cellulose is a complex polysaccharide consisting of a covalent structure as a β (1-4)-linked D-glucan with a DP of more than 10,000 in secondary walls and 2000–6000 in primary cell walls. The formation of hydrogen bonds during the interactions of parallel glucan chains leads to the synthesis of newly cellulose microfibrils conferring the formation of a strong and extensible three-dimensional network. Further, pectic polysaccharides consist of polysaccharides rich in α-D-galacturonic acid (GalA) residues, in which varying proportions of the acid groups are present as methyl esters. As complex macromolecules, they can be composed of 17 different monosaccharides comprising more than 20 different linkages. The major types of pectic polysaccharides backbone are homogalacturonan (HG) and rhamnogalacturonan I (RGI). They, in the primary cell wall, are characterized by certain amounts of neutral sugars present as side chains. The most abundant neutral sugars are arabinan and galactan [114,116,117,118,119,120,121]. Hemicelluloses as another category of NSPs, are characterized by β-(1→4)-linked backbones of sugars with an equatorial configuration, a DP of between 150 and 200, and can be extracted with alkaline treatment. They comprise xyloglucans, xylans, mannans, and glucomannans [116,122,123]. The major sources of carbohydrates in the human diet are cereals, tubers, legumes, pulses, fruits, vegetables, fungi, algae, seaweeds, guar, etc. [115].

##### Amino Acid Derivatives

Proteins are the essential components of tissues in all organisms [124]. The nutrition chemistry, in its investigations, has emphasized the significance of amino acids as the fundamental factors in all concerns in which the proteins have been involved over the years [125]. The three-dimensional structure of proteins affects their function [126]. François Magendie, French experimental physiologist, in 1816 showed that dogs fed foods, containing protein, remained healthy. Whereas dogs fed, containing only fat or carbohydrates, lost weight and developed a corneal ulcer for two weeks and after a month they died. These observations have identified the protein as a specific essential dietary component [95,127]. The nutritional value of dietary protein has been raised as, during hydrolyzation by proteases and peptidases, it generates amino acids, dipeptides, and tripeptides in the lumen of the gastrointestinal tract. A protein contains various amounts of 20 different amino acids which are linked to each other via amide bonds, the so-called peptide bonds [124]. Amino acids, as organic substances that provide nitrogen, hydrocarbon skeletons, and sulfur, have played a significant role in the nutrition and health maintenance of humans and animals. Almost all amino acids have an asymmetric carbon and show optical activity. Glyceraldehyde has been used as a reference to define the absolute configuration of amino acids such as L- or D-isomers. Even though there are more than 100 amino acids in nature, only 20 of them are considered as building blocks of protein. These amino acids have an amino group (^+^NH_3_), a hydrogen atom, a carboxyl group (COO^−^), and a side chain (R) attached to the central α-carbon. Amino acids are classified, based on nitrogen balance, as nutritionally essential or non-essential for humans and animals. Nutritionally non-essential amino acids are synthesized by animals or humans and are not necessary to be provided from the diet. Contrarily, the essential ones cannot be synthesized by an animal, and therefore must be ingested with feed. There are nine essential amino acids, of the 20 standard protein amino acids, including L-leucine, L-valine, L-threonine, L-isoleucine, L-methionine, L-lysine, L-phenylalanine, L-histidine, and L-tryptophan [128,129,130,131]. Amino acids that are impressive regulators of key metabolic pathways to improve health, maintenance, growth, immunity, and reproduction of organisms, have led to the development of the functional amino acids concept. The major sources of amino acids are several natural plant proteins and animal products [130].

##### Microbes and Minerals

In recent years, the great demands, for augmenting the value of nutraceuticals to cure diseases, have notably affected the signs of the progress of nutraceuticals production via metabolic engineering of microbial-based platforms [132]. Some of the microbes, among trillion others that colonize the human body, can potentially be beneficial or harmful. An imbalance of them may cause several diseases, therefore, probiotic and prebiotic supplements may be effective to prevent such conditions. According to a joint Food and Agriculture Organization of the United Nations (FAO) and World Health Organization (WHO) in 2001, probiotics are defined as, “*Live microorganisms which, when administered in adequate amounts, confer a health benefit on the host*.” Afterwards, the study of beneficial bacteria revealed the nondigestible food ingredients, called prebiotics, which stimulate the activity and the growth of these bacteria in the intestinal tract. Human origin, non-pathogenic quality, stability in acid and bile, resistance to technological processes, production of antimicrobial substances, the modulation on the immune system, the persistence within the GI tract, the influence on metabolic activities are some criteria, among others, that a microbe must accomplish to be classified as probiotic [132,133,134]. The most known probiotics are the lactic acid bacteria *Lactobacillus acidophilus*, *Lactobacillus casei*, and bifidobacterial types. The main sources of them are yogurts and other dairy products such as buttermilk, frozen desserts, milk powder, and acidophilus milk and some non-dairy products such as fruits, vegetables, legumes, and cereals [135,136]. Furthermore, dietary essential minerals are crucial components to uphold several bodily functions [137]. It has been established that several mineral elements are indispensable for normal nutrition constituting approximately between 4% and 6% of body weight. Mineral nutrition has been more important than vitamin nutrition since the body, using some minerals, can replace the lacking vitamins. Whereas, the opposite is hopeless [138]. There are 20 essential minerals for humans divided up into major minerals and trace minerals. Sodium, chloride, phosphorus, potassium, magnesium, calcium, and sulfur are major minerals. On other hand, the trace minerals comprise iron, zinc, iodine, selenium, copper, manganese, fluoride, chromium, and molybdenum. Milk and dairy products are considered to be significant sources of the daily intake of essential minerals [139,140,141].

#### 2.4.3. Nutraceuticals Based on Mechanism of Action

Concerning specific therapeutic properties, nutraceuticals are known for anti-inflammatory, anti-microbial, anti-oxidant, anti- hypercholesterolemic, anti-aggregate, anti-hypertensive, anti-carcinogenic, osteogenetic, or bone protective properties, etc. [84,94].

##### Nutraceuticals and Health Benefits

The establishment of a vibrant nutraceutical research community is necessary to spread scientific knowledge about nutraceuticals. This has enabled the creation of the established nutraceuticals from the potential ones and offered the delivery of their enormous benefits across the globe. The reflection of the continuous research, market expansions, and consumer interest is made by the constant changing list of nutraceuticals being investigated [142]. As a result of the unhealthy diet, tobacco use, harmful use of alcohol, irregular sleeping habits, and a lack of daily physical exercise, there are countless global health problems related to the advancement of diabetes mellitus, cardiovascular morbidity and mortality, chronic respiratory diseases, metabolic syndrome, and cancer. They may be summarized with the term “Chronic Non-Communicable Diseases” (NCDs) as a distinguishing feature of lifestyle diseases. Fighting of the aforementioned has evoked several arguments on nutraceuticals efficiency [92,143,144,145]. The challenge has been to define the interrelationship between the disease and nutrient [104]. Many industries such as foods, herbals, and pharmaceutical manufacturing have evaluated nutraceuticals as beneficial products related to the cure of many health troubles. In addition, the nutraceutical safety and their side effects such as allergic reactions, cardiac arrhythmias, insomnia, their interactions with other nutraceuticals and therapeutic drugs, etc., are marked [146]. Although the adverse side effects of nutraceuticals are usually minimal compared to synthetic drugs [104], their use must be regulated and controlled with experimental assessment or clinical trials [146]. According to the World Health Organization (WHO), lifestyle diseases are one of the most momentous challenges of twenty-first century medicine. The statistics in 2016 have shown that 40.5 million (71%) deaths are due to NCDs among 56.9 million of total premature deaths, 17.9 million (44%) deaths are due to cardiovascular pathologies, 9 million (22%) to cancer, 3.8 million (9%) to chronic respiratory diseases, and 1.6 million (3%) to type 2 diabetes. The main concern is that the toll of deaths can reach 52 million in 2030 if the growth rate continues so [143].

According to the literature described in this subsubsection (2.4.3.), some prominent evidence in the new era of the twenty-first century have shown the enormous growing of nutraceuticals as potent therapeutic supplements. The preventive therapeutic efficacy of new nutraceuticals can be practically extended if their miraculous health benefits are investigated [147]. Therefore, in this review, various nutraceuticals applications will be considered focusing on the rise of more recent afflictions such as cancer, diabetes, neurological, cardiovascular, and chronic diseases, which have emerged as public health problems in many countries [148].

##### Anti-Microbial Activity

The role of nutraceuticals in the inhibition of microorganisms and alteration of bacterial populations is still implied despite the incompleteness of information. The products such as aloe, goldenseal, St. John’s wort, garlic, zinc oxide, echinacea, and zinc gluconate, are studied for their antibacterial activity. As the gram-positive bacteria, Staphylococcus aureus ATCC 29213 is used whereas Escherichia coli ATCC 25922 is used as the gram-negative bacteria. It has been observed that some products can be selective agents in the development of antibiotic resistance and lose their antibacterial properties quickly [12]. Therefore, their effectiveness needs further investigation. Phenolic compounds can also be used as antibiotics, antidiarrheal, or antiulcer agents [149], being involved in various physiological processes of plants and plant defense mechanisms against microbial infections [150].

##### Anti-Oxidant Activity

**Figure 5:**
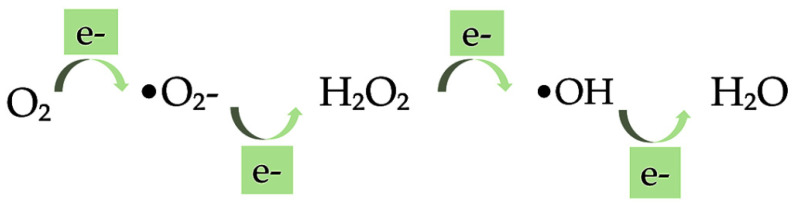
Formation of superoxide radical (•O_2_−), hydrogen peroxide (H_2_O_2_), hydroxyl radical (•OH), and water by stepwise, univalent reductions of molecular oxygen.

The significant and important application of oxygen in clinical medicine can also bring certain toxic effects [151]. Oxygen is considered a double-edged sword; it has promoted and destroyed life for two centuries. Liebig, in 1842, highlighted that toxic oxygen, capable of burning up all the tissues, can be removed from the organism by carbon and hydrogen, that acts as antioxidants, in food. He believed in this since in starvation there is no food to remove the oxygen, and therefore the particles of the brain begin to undergo the process of oxidation. On the other hand, carbon and hydrogen-rich food, considered as antioxidant, by reacting with oxygen can inhibit the destructive influence of oxygen in the tissue [152]. The development of major diseases is supported by oxidation processes that occur naturally in the human body [153]. Therefore, oxygen toxicity has emerged as one of the most fundamental phenomena in biological sciences. Gerschman, in 1954, formulated a general theory of oxygen toxicity describing the oxygen-induced damage that is caused by free radical intermediates. The oxidizing free radicals are generated in excessive amounts when the living organisms are exposed to the increased pressure of oxygen [152,154]. Free radicals and other reactive oxygen species (ROS) are considered potentially harmful agents but are also known to produce various cellular structures and to fight pathogens [155,156]. The superoxide •O_2_−, hydroxyl radical •OH, and hydrogen peroxide H_2_O_2_ are free radicals produced by metabolic reactions in the human body, as shown in Figure 5. These are molecules with one or more unpaired electrons. ROS refer to any free radical containing oxygen but can also include non-free radical species such as hydrogen peroxide H_2_O_2_, ozone O_3_, singlet oxygen ^1^O_2_, hypochlorite ^−^OCl, and peroxynitrite ONOO^−^. In comparison with non-radicals, the free radical reactions result in new radicals leading to chain reactions.

The •O_2_− radical is produced by the first one-electron reduction of molecular oxygen. It can operate as an important second messenger in the cell even though its reactivity and toxicity are low. Further, the H_2_O_2_ falls as a result of the dismutation of •O_2_−. Owing to high reactivity, H_2_O_2_ forms the •OH when it reacts with partially reduced metal ions. The •OH is considered the most important radical, among others, with a high impact on the cell damage as it can directly evoke DNA damage. The general mechanism that the free radicals can be oxidized to oxygen and reduced to water protects the biological systems from the potential hazards of those radicals. The backbone of the cellular antioxidant defense system is composed of the antioxidant enzymes superoxide dismutase (SD), catalase (CAT), and glutathione peroxidase (GPX). The dismutation of •O_2_− to H_2_O_2_ is catalyzed by SD, whereas the detoxification of H_2_O_2_ is made by CAT. In addition, GPX, using reduced glutathione (GSH) as the electron donor, reduces organic hydroperoxides and H_2_O_2_. GSH is a tripeptide with a reactive sulfhydryl group, and has multiple effects regarding the antioxidant defense. These effects comprise its action as a scavenger of free radicals such as •O_2_−, •OH and lipid hydroperoxides, as a substrate for the antioxidant enzyme GPX and in the direct repair of oxidative DNA lesions. As the effects of oxidative stress on human health are considered a serious issue [157], nutraceuticals with antioxidant activities have received attention [96]. The antioxidant activity of nutraceuticals is affected by their chemical structure [158]. Dietary components with important antioxidant functions comprise ascorbate, α-tocopherol, β-carotene, linoleic and linolenic acids, copper, manganese, zinc, selenium, and cysteine [152]. The clinical trials on the role of antioxidants have mainly focused on several compounds, such as carotenoids, vitamins C and E [155]. Carotenoids are efficient antioxidants involved in the scavenging of singlet molecular oxygen and peroxyl radicals. The physical quenching enables the direct transfer of energy between carotenoids and ^1^O_2_. The ground state oxygen and a triplet excited carotene are yielded as a result of the transfer of ^1^O_2_ energy to the carotenoid molecule. Further, the carotenoid dissipates its energy returning to the ground state throughout its interaction with the surrounding solvent [159]. β-carotene, among the various carotenoids, is an effective quencher of singlet oxygen preventing lipid oxidation (Table 1). 

Vitamin E or α-tocopherol prevents membrane-mediated effects of oxygen free radicals because it efficiently protects biological membranes from lipid peroxidation, as it is described by a nonenzymatic reaction (Table 2) [160].

The reduction of vitamin E radical must go further by its interaction with ascorbic acid or vitamin C (Table 3). The ascorbate is considered an antioxidant because of its direct participation in the scavenging of the “activated oxygen”.

**Figure 6:**
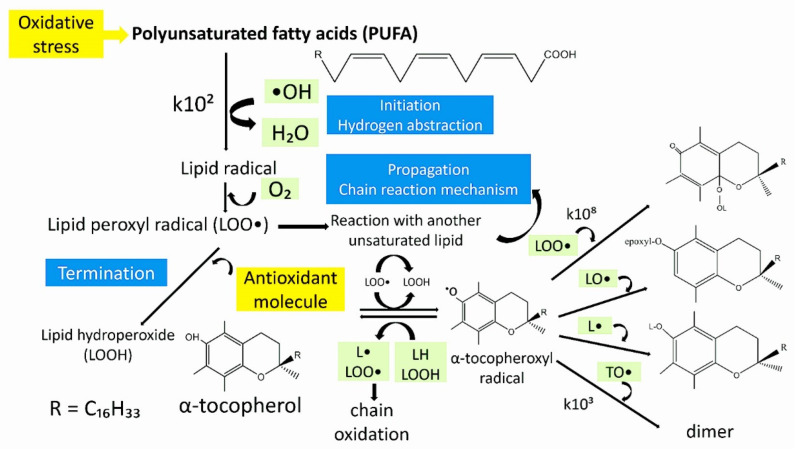
The reaction of α-tocopherol during the autoxidation of unsaturated lipids. LOOH-lipid hydroperoxide; LO•-lipid-alkoxyl radical; L•carbon-centered lipid radical; LOO•-lipid-peroxyl radical; TO•-α tocopheroxyl radical; k-rate constant in M^−1^ s^−1^.

The combination of both β-carotene and α-tocopherol inhibits the lipid peroxidation more efficiently than their individual use [159]. The reaction scheme of α-tocopherol during the autoxidation of polyunsaturated fatty acids is presented in Figure 6. Initiation, propagation, and termination, are three stages that the autoxidation as a chain reaction proceeds. The carbon-centered lipid radical or an alkyl radical is produced in the initiation step by the abstraction from a polyunsaturated fatty acid moiety. The alkyl radical, in the propagation step, reacts with molecular oxygen at a very high rate, giving a peroxyl radical. The peroxyl radical is a chain-carrying radical and, therefore, can attack another polyunsaturated lipid molecule. Although the initial peroxyl radical is converted to a hydroperoxide, a new alkyl radical is produced and rapidly converted to another peroxyl radical. The chain reaction continues until inactive products are formed because of the combination of the chain-carrying peroxyl radical with another radical. α-Tocopherol inhibits the propagation step as a chain-breaking antioxidant. A peroxyl radical, after receiving the phenolic hydrogen atom by α-tocopherol is converted to a hydroperoxide. The tocopherol radical, incapable of continuing the chain, is formed. Further, it is removed from the cycle by the reaction with another peroxyl radical to form an inactive and non-radical product. The measure of the antioxidant efficiency of α-tocopherol is made by the rate at which it reacts with peroxyl radicals [161]. Phenolic compounds are considered as strong antioxidants that complement the functions of enzymes and antioxidant vitamins as a protection against oxidative stress caused by excess reactive oxygen species (ROS) [99]. Their elevated capacity is related to scavenging free radicals [162]. Flavonoids, an important class of phenolic compounds, suppress reactive oxygen formation during the antioxidant mechanism, by scavenging reactive species, inhibiting enzymes, chelating trace elements involved in free-radical production, protecting and up-regulating antioxidant defenses [157]. Therefore, they are among the most efficient antioxidant molecules [162]. Tannins [158], terpenes [163], sterols [153], and fiber [164], have also shown antioxidant activities. Further, the excessive production of ROS can also cause many other disorders such as hypertension, inflammation, cataract, cardiovascular disease, diabetes, cancer and neurodegenerative diseases, and osteoporosis emphasizing the significance of phenolic compounds in the inhibition of the ROS formulation [101,165].

##### Anti-Hypertensive Activity

Hypertension is known as one of the most frequent chronic medical conditions in the developed world. It is also considered a major hazard factor for coronary heart disease, stroke, congestive heart failure, and renal disease. Hypertension is a result of the environment-genetics interaction. Inflammation, subsequent gene expression, oxidative stress, nutrient–gene interactions can positively or negatively affect human vascular biology [166]. As hypertension is mainly treated with anti-hypertensive drugs, the use of blood pressure-lowering nutraceuticals is of great interest [167]. Vitamin D3, vitamin C, vitamin B6, amino acids (taurine, arginine, carnitine), chlorogenic acids, melatonin, coenzyme Q10, quercetin, probiotics, and resveratrol are some nutraceutical supplements with an influence in the treatment of hypertension [166].

Chlorogenic acids can be found in fruits, plants, and vegetables such as coffee beans, tomatoes, apples, etc. The mechanism of chlorogenic acids for reducing blood pressure is well-known. Firstly, the consumption of chlorogenic acid is important because it is an antioxidant. The superoxide radical causes hypertension by forming peroxynitrite in vascular walls through the destruction of nitric oxide (NO). NO bioavailability can be increased by inhibiting the reactive oxygen species. This step generates enzymes such as xanthine oxidase and DAD(P)H, and reduces the formation of peroxynitrite. Secondly, the protective role of chlorogenic acid in eNOS causes the induced anti-hypertensive activity. The antihypertensive response of chlorogenic acid in hypertensive rats (SHR) is blocked by the addition of the N(G)-nitro-L-arginine methyl ester (L-NAME) as a NOS inhibitor. As it is known that the blood pressure is adversely associated with the plasma level of NO metabolites, chlorogenic acid intake increased the urinary NO metabolites in SHR. The effect of blood pressure reduction is mediated by inhibiting angiotensin-converting enzyme (ACE) activity, modulating nitric oxide (NO) production, scavenging free radicals, and improving endothelial function through the products that naturally contain the nutraceuticals [168].

Vitamin D is produced by the non-enzymatic conversion of provitamin D3 to previtamin D3 [169]. Vitamin D receptors are found in the kidney (juxtaglomerular cells), leukocytes, cardiac myocytes, and vascular smooth muscle cells of the human body. A study demonstrated that vitamin D directly suppresses renin synthesis. This is because of the reduction in renin mRNA transcription in the kidney. The plasma renin appears because of vitamin D deficiency. Further, vitamin D alters the epidermal growth factor receptor function and, therefore, inhibits the proliferation of vascular smooth muscle cells. Vitamin D suppresses pro-inflammatory cytokines, reduces asymmetric dimethyl arginine, improves endothelial function and arterial elasticity, increases nitric oxide (NO), and decreases vascular smooth-muscle hypertrophy. For optimal blood pressure lowering effects, it is recommended a vitamin D level of 60 ng/mL [170].

Resveratrol is another nutraceutical that presents an anti-hypertensive effect [171] and can be found in red grapes, and in plants that can survive harsh environmental conditions [172]. Resveratrol improves endothelial dysfunction, prevents the uncoupling of endothelial nitric oxide synthase (eNOS), increases the flow-mediated vasodilation in a dose-related manner, and blocks the effects of angiotensin II [173].

Lycopene can be found in tomatoes, red grapefruits, watermelon, etc. [174]. A study presented a significant blood pressure reduction of 5.4/3 mmHg over six weeks after the administration of standardized tomato lycopene extract [166]. Further, the spontaneously hypertensive rats (SHR) were studied. In this strain, the hypertension was progressively increased over time, and a four-week of lycopene supplemented diet was employed. An effective blood pressure reduction, in both young and adult rats, was observed. This research supported the effectiveness of lycopene in hypertension prevention [175]. This is because of the anti-hypertensive effects of lycopene inhibiting angiotensin-converting enzyme (ACE), reducing oxidative stress that is induced by angiotensin-II, and transversally enhancing the production of nitric oxide in the endothelium [174].

##### Anti-Inflammatory Activity

The various classes of terpenoids demonstrate health benefits through their connection with key molecular players in animal and human physiology, action as immunostimulants, antioxidant activity booster, blood coagulation hemostasis modulator, related to anti-cancer, anti-malaria, anti-bacterial and anti-viral activities. Terpenoids also modulate transcription factors like the nuclear factor kappa B (NF-κB) related to the regulation of a cascade of events in inflammatory pathways that cause various chronic diseases such as cardiovascular disease, diabetes, Alzheimer’s, etc. Scientific studies have shown that several terpene-based volatile compounds occurred in plant essential oils including compounds such as α-pinene, β-limonene, p-cymene, linalool, β-phellandrene, and terpinenes, can have anti-inflammatory, anti-oxidant effects, and can cross the blood-brain barrier and treat the Alzheimer’s disease [176].

Fatty acids can affect cellular functions and physiological responses due to their principal roles as energy sources and membrane constituents [105,106]. Fatty acids serve as substrates for the biosynthesis of biologically active lipid mediators and play direct roles in cell signaling that influences gene expression. The responsiveness, and functionality of the cells and tissues can be modified through the mix of complex lipids and their constituent fatty acids. This phenomenon is well-defined for immune, metabolic responses and inflammatory, cardiac and neurological function, platelets, etc. The inflammatory effect of the fatty acids is the most considered. A well-functioning immune system is crucial to human health and serves to protect the host from the effects of infectious agents that exist in the environment. An immune response to host tissue is generated when the immune system recognizes the host antigens as “non-self” rather than as “self”, leading to tissue damage as a characteristic of so-called chronic inflammation [177]. Therefore, the inflammatory process appears when the human body tries to fight infection or to repair damaged tissue, leading to the progression of some chronic diseases such as rheumatoid arthritis, inflammatory bowel diseases, asthma, cardiovascular disease, neurological disease, type-1 diabetes, cancer, oncologic or endocrinologic diseases. Fatty acids have a high impact on human health by influencing the appearance or evolution of those diseases [105,109]. Omega-3 and omega-6 PUFAs are the most important fatty acids, and their balance can be important in determining the seriousness and development of the diseases. The inflammatory process can be generated by a high intake of omega-6 PUFAs, particularly arachidonic acid. Conversely, long-chain omega-3 PUFAs are potentially potent anti-inflammatory agents that decrease the expression of adhesion molecules and the production of inflammatory mediators such as eicosanoids, cytokines, and reactive oxygen species. The anti-inflammatory effect of omega-3 PUFAs is related to their direct action attributed to their capability of competing with arachidonic acid or their indirect action of affecting the transcription factors or nuclear receptors responsible for inflammatory gene expression [109,178,179]. The data from experimental and clinical studies [177] have presented the long-chain ω-3 PUFAs as potential therapeutic agents for inflammatory and autoimmune diseases [180]. The most valuable long-chain omega-3 PUFAs are eicosapentaenoic acid (EPA) and docosahexaenoic acid (DHA). Their beneficial effects regarding the cardiovascular diseases interfere with the broad spectrum of anti-arrhythmic, lipid-lowering, anti-thrombotic and anti-inflammatory properties [109,181,182,183]. Moreover, the direct role of ω-9 fatty acids, in comparison to the ω-3 and ω-6, in inflammatory pathways has been unclear [113] until several in vivo experiments demonstrated it. It has been related to the decreasing production of proinflammatory cytokines such as tumor necrosis factor-alpha (TNF-α) and interleukin 1-beta (IL-1β), and with enhancing production of anti-inflammatory cytokine such as interleukin-10 (IL-10) [184]. Cytokines are a group of cell-derived polypeptides that participate in a complex network of interactions exhibiting both negative and positive regulatory effects in growth, development, or activity of various target cells [177,185]. The ROS-scavenging activity of β-carotene and lycopene has enabled their use as anti-inflammatory substances [186].

##### Anti-Hypercholesterolemic Activity

The high levels of lipids such as cholesterol, triglycerides, and fat phospholipids in the blood cause the pathological condition called hypercholesterolemia. A prolonged increase in insulin levels, as well as a high level of O-GlcNAc (O-linked β-N-acetylglucosamine), can affect hypercholesterolemia, and lead to dyslipidemia [187]. The development of cardiovascular diseases (CVDs) is more likely to be present in patients with hyperlipidemia [188]. The oxidative stress, induced by reactive oxygen species (ROS) [189], can develop the CVDs and atherosclerosis as previously described in the subsubsubsection of antioxidant activity that oxygen species are likely to be involved in the pathophysiology of many human diseases, such as CVD, cancer, etc. In CVDs, oxidative stress alters the gene expression. Further, the transcription factor activity, particularly NF-κB, activator protein-1 (AP-1), and the peroxisome proliferators-activated receptor (PPAR) family of transcriptional activators are modulated by increased ROS levels. The oxidative modification of low-density lipoprotein (LDL) is one of the first events, in CVDs, that appeared as a consequence of increasing ROS generation [190]. The abnormally low uptake of low-density lipoprotein (LDL) by the liver is caused by a genetic defect of the low-density lipoprotein receptor (LDLR), leading to familial hypercholesterolemia (FH) [191]. It is found that 20% of patients are diagnosed with FH, and only a minimum of them have received the appropriate treatment [192]. Therefore, hypercholesterolemia can be ameliorated by having an appropriate drug treatment, and adequate lifestyle [187]. Red yeast rice, berberine, plant sterols and stanols, dietary fibers, polyphenols, flavonoids, and apple polyphenolic extract are some of the nutraceuticals which claim to have a cholesterol-lowering effect. Plant sterols and stanols are found to inhibit the absorption of cholesterol if they are taken at a dose of g/day. At this dose, they lower LDL-cholesterol (LDL-C) levels by 8 to 10%, and reduce plasma triglycerides between 6 and 9%. However, the effective use of plant sterols and stanols, on total cholesterol and LDL cholesterol, has been observed [193,194]. Further, some bioflavonoids are found to lower cholesterol levels but the information about their bioavailability, presence of contaminants in their original vegetal matrix, and their unwanted side effects, is still lacking [194].

##### Anti-Aggregate Activity

Another factor that causes the cardiovascular disorders has been the blood platelet activation. The progression of hemostasis, atherosclerosis, and other diseases of the cardiovascular system has been linked to the dysregulation of blood platelet activity. Blood platelet aggregation has been a result of the modulation of platelet function. Platelets, or thrombocytes, are anucleate cells, between 2 and 4 μm in greatest diameter, produced by megakaryocytes. Before being eliminated by the liver and spleen, they circulate in the human bloodstream between seven and ten days [195,196]. Nutraceuticals, as antiplatelet agents, are found to have beneficial effects. As resveratrol has been well-known for its various biological activities, one study [197] investigated the effect of cis-resveratrol on platelet aggregation. The 4-channel aggregometer was used to perform the platelet aggregation. Acid-citrate-dextrose (1:6) was used as an anticoagulant to collect the blood from the abdominal aorta of ether-anesthetized rats. The indicated concentrations of cis- or trans-resveratrol were used to incubate the platelets obtained. Whereas the sub-maximal concentrations of thrombin, collagen, or ADP were used to induce aggregation. This aggregation was further suppressed by both, cis-resveratrol and trans-resveratrol. It is found that 3.6 μg/L resveratrol reduces collagen-induced platelet aggregation by 50.3%. Resveratrol interferes with platelet aggregation by inhibiting Ca^2+^ influx that is essential for platelet aggregation [198]. The modulation of nitric oxide (NO) is another anti-platelet aggregation mechanism of resveratrol. Resveratrol promotes NO production via increasing eNOS expression and activity [199]. This activity is beneficial because NO maintains the vasculature homeostasis, and regulates intracellular signaling pathways. NO limits the thrombotic process by decreasing endothelial cell adhesion and inhibiting platelet aggregation. NO inhibits platelet aggregation by upregulating cyclic guanosine monophosphate, reducing dimerization of integrin αIIbβ3, and hindering von Willebrand factor (VWF)-mediated platelet adhesion [200].

##### Anti-Carcinogenic Activity

**Figure 7:**
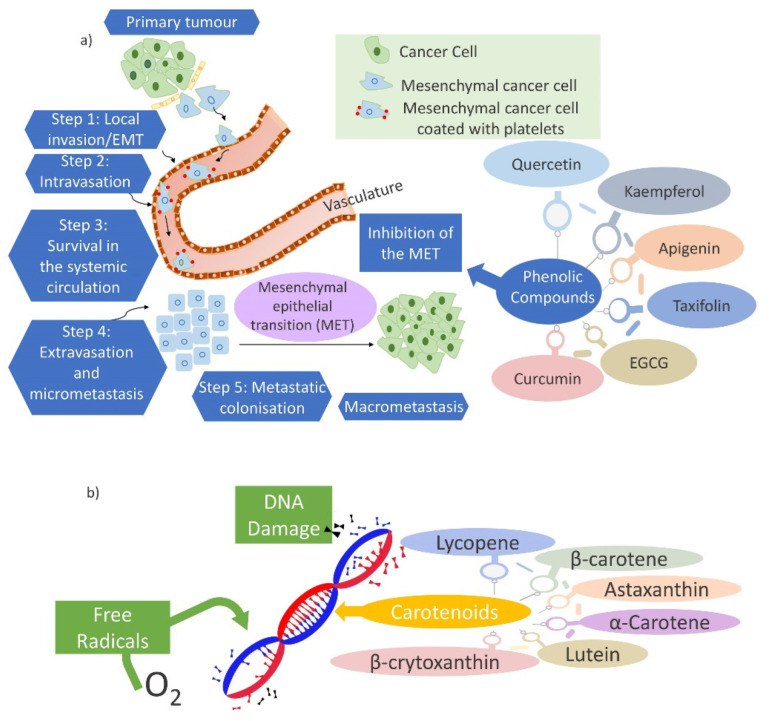
(**a**) The inhibition of the epithelial–mesenchymal transition (EMT) by phenolic compounds. The EMT is a critical part of cancer metastases and consists of the following key steps: Step 1 describes the subjecting of epithelial cancer cells to EMT which cells may then intravasate into the systemic circulation (Step 2). These EMT-induced cells must survive in the circulation (Step 3) before reaching the target. Then, the cells that reach Step 4 must extravasate into the tissue parenchyma upon reaching the target organ site and form micrometastases. At the end (Step 5), the mesenchymal–epithelial transition (MET) activation, another critical event for the metastasis of carcinomas, is required as a subsequent development into potentially life-threatening macrometastases [211,212]. (**b**) The prevention of free radical-induced damage to cellular DNA by carotenoids.

The treatments of assorted forms of cancer, leading cause of death worldwide, are poorly controlled and have serious side effects. ROS are likely to be involved in the pathophysiology of many human diseases. The imbalance and high level of free radicals such as ROS and reactive nitrogen species (RNS) can affect cancer development. Chemotherapy causes undesired side effects, therefore, diet-related agents are a category of cancer chemopreventive agents that have generated much attention and interest during recent decades [201,202,203]. However, the physiological relevance of these agents is uncertain [204]. The phenolic compounds are powerful antioxidants that have been used as alternative treatments for cancer [201]. Several studies showed that quercetin, luteolin, kaempferol, apigenin, taxifolin, (-)-epigallocatechin-3-gallate (EGCG) [205], ethanolic extracts of Curcuma rhizome and Zingiber rhizome [206], curcumin [207], are some natural phenolic compounds exhibiting anticancer effects. These compounds affect human cancer cell lines by protecting or reducing the number of tumors and their growth [208]. Their anticancer efficacy may be due to the inhibition of the epithelial-mesenchymal transition (EMT) (Figure 7a), as one of the main pathways employed in cancer development and metastasis, in cancer cells. The phenolic compounds can also prevent cancer initiation, relapse, and metastasis [209]. Further, carotenoids play a significant role in cancer prevention. The effect of a carotenoid on cellular differentiation and proliferation, the prevention of free radical-induced damage to cellular DNA (Figure 7b), and other molecules from the antioxidant function, and the enhancement of immune surveillance in tumorigenesis from the immunomodulatory effects, are several mechanisms related to the cancer prevention of carotenoids. β-carotene, lycopene, lutein and zeaxanthin are the most studied carotenoids [210]. Aside from the beneficial effects, β-carotene and other carotenoids have been found to increase cancer risk but the level of evidence is limited. The effectiveness of chemopreventive agents has been related to the determination of the proper effective dose. For instance, humble levels of folic acid supplementation suppress the development of cancer and vice versa [202]. Furthermore, a high dose of β-carotene can expand the risk of lung cancer [203,210].

##### Bone Protective Activity

Bone is a dynamic tissue in a continuous cycle of bone resorption followed by bone formation. Established bone is degraded by osteoclasts through adherence, acidification, and proteolytic digestion. Then, osteoanabolic therapies are used for new bone formation. A role in promoting bone health is shown by the dietary intake of fruits and vegetables, more specifically by the polyphenols that describe the physiological effects associated with bone material density and bone metabolism [213,214]. Osteoporosis and osteopenia, corresponding with a decrease in bone formation, can be prevented by lifestyle modifications. Adequate nutraceutical supplementation such as calcium intake supplemented by vitamin D, magnesium, potassium, copper, resveratrol, green tea, prebiotics and probiotics, polyunsaturated fatty acids, melatonin, have shown promising results for the management of osteoporosis [215,216].

## 3. Delivery Systems for Nutraceuticals

**Figure 8:**
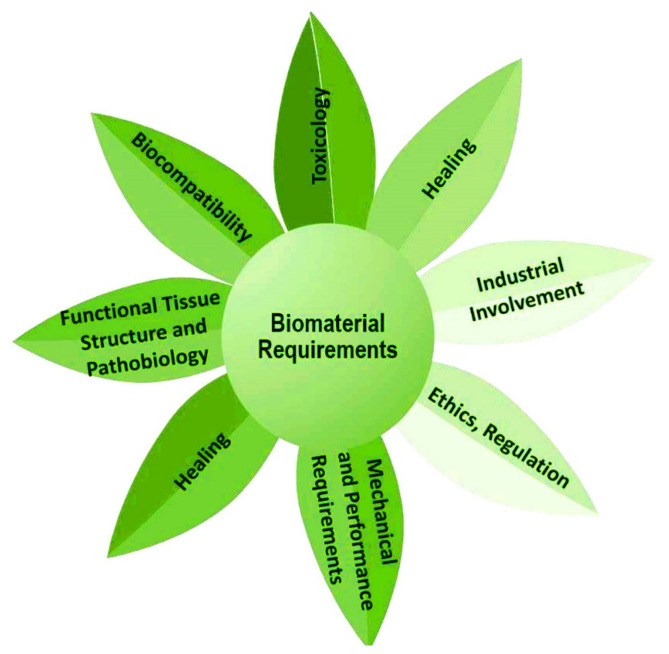
The essential requirements involved in biomaterial design for nutraceuticals delivery.

A nutraceutical has not always met the requirements to achieve the therapeutic purpose [10]. Therefore, the systems for their delivery must be designated to produce products that have consistent quality attributes. The “biomaterials science” is considered as the main focus toward the development of materials, tailored to a specific application, that can elicit highly precise reactions with proteins and cells. The biomaterials synthesis, characterization, testing, optimization, and the biology of host-material interactions are highlighted during the most intense investigation. The biomaterial must accomplish various requirements such as toxicology, biocompatibility, functional tissue structure, and pathobiology, mechanical and performance requirements, healing, industrial involvement, regulation, etc. (Figure 8) [217]. Nanotechnology is advantageous for manipulating the properties and structures of materials at the nanometer scale, and therefore has opened up new opportunities for numerous applications in biotechnology, molecular biology, medicine, environmental science, etc. [218]. The field of nanotechnology, through the efficacy of nano-drug delivery systems, is contributing to every walk of life improving the bioavailability, biocompatibility, solubility, drug loading efficacy, and surface modifications of bioactive and chemical molecules [219]. The application of nanotechnology in health care is extensively adopted as a robust driver of biomedical novelty [220].

### 3.1. *Advisable Features of Delivery Systems*

#### 3.1.1. Encapsulation and Controlled Release Capacity

To deal with the limitations of the aforementioned nutraceuticals, encapsulation technology has stood out for decades [221]. The encapsulation requires essential considerations such as stability, the inherent physicochemical characteristics, the interactions between the active component and the matrix, etc. [222]. Bioactive molecules such as isoprenoid derivatives, fatty acids, phenolic substances, structural lipids, carbohydrates, aminoacid derivatives, microbes, and minerals, must be encapsulated before their delivery into a system. The incorporation of the bioactive component in a solid or liquid matrix, the dispersion or spraying of liquid in case of a solid matrix solution, and the stabilization of the system through a physical, chemical, or physicochemical process, are the stages that comprise the encapsulation process. It helps that the bioactive substances are protected from adverse environments, thus, improving their bioavailability [223]. The surface release, diffusion through the swollen matrix, and erosion of the matrix are the three steps that result in the release of the bioactive from encapsulants [224]. The release kinetics can be controlled by the diffusion of a drug molecule through the carrier matrix, and it is desirable to develop drug carriers that provide the sustained or controlled release of the drug with a low dosing frequency. Drug release from carriers is affected by various factors including the composition (drug, polymer, and additives), their ratio, physical and/or chemical interactions among the components, and the methods of preparation [225].

#### 3.1.2. Solubility

The foremost issue encountered with the formulation development of new drugs is the low aqueous solubility [226]. Poor water solubility is a significant risk factor in low oral absorption [227] because the molecular dispersion of a drug is necessary for its absorption across biological membranes. The drug, firstly, must be dissolved within the gastrointestinal tract (GIT) [228], and then absorbed. Subsequently, it reaches the systemic circulation that is important for producing the desired pharmacological response after oral administration. The low solubility of many drugs is a major obstacle to the development and the large-scale production of oral solid dosage forms. Accordingly, the major goals of designing and developing new drugs are the improvement of the solubility and the determination of its negative influence on the drug absorption, bioavailability, stability, and therapeutic effect [229,230].

#### 3.1.3. Bioavailability

As previously mentioned, poor bioavailability is the major challenge in designing oral dosage forms. First-pass metabolism, aqueous solubility, dissolution rate, drug permeability, presystemic metabolism, and susceptibility to efflux mechanisms, are various factors on which the oral bioavailability depends [226]. The study of nutraceuticals bioavailability is an important [231], and an urgent necessity, because of the growth of health challenges, and rapid population [232]. β-carotene, vitamin E, various polyphenols such as phenolic acids, stilbenes, flavonoids, lignans, etc., are slowly absorbed, and therefore have a limited bioavailability [233]. Bioavailability is a property of the drug alone and its delivery systems. Low bioavailability of the drug on its therapeutic use can be considered safe for oral administration because it can be administered in excess without any adverse effects. To increase the bioavailability, the development of powerful drug delivery systems, for surviving the harsh acidic environments of the stomach and rising absorption through the intestinal wall, is considered [232,234].

Bioaccessibility (B*), absorption (A*), and transformation (T*), are the three main stages that the studies on the nutraceutical bioavailability climax. Bioaccessibility, the first step is defined as the fraction of ingested nutraceutical that becomes accessible for absorption through the epithelial membrane of the intestine, whereas absorption, the second step, comprises biocomponent absorbed at the level of the gastrointestinal tract (GIT) epithelial cells. Further, transformation, the third step, describes the chemical or biochemical transformations in the GIT fluids during their digestion and metabolism in the liver [235].

### 3.2. Delivery Systems Design

As the properties of nutraceuticals such as encapsulation, release capacity, bioavailability and biocompatibility, solubility are challenges still to be overcome, nanotechnology has involved the design and development of organic and inorganic materials at the nano scale, with tailor-made physical, chemical, and biological properties [220]. Many scientists, when designing various delivery systems, utilized approaches such as protection of labile compounds, extension of gastric retention time, controlled/delayed-release, lymphatic uptake facilitation, intestinal permeability enhancement, and modulation of metabolic activities for an optimum nutraceutical delivery system [236]. Lipid, surfactant, and biopolymer-based delivery systems are widely explored as carriers for drug delivery.

The strategies needed for the nutraceutical delivery systems are obtained including the strategies based on drug delivery [237]. Lipid-based delivery systems include liposomes, nanoemulsions, solid lipid nanoparticles (SLNs), niosomes, nanostructured lipid carriers (NLCs), and self-emulsifying drug delivery systems (SEDDSs) [238]. In contrast, surfactants tend to self-assemble in aqueous solutions into micelles, bilayers, vesicles, liquid crystals, and reverse micelles. Some of the lipid-based delivery systems are unstable systems over time or when they are exposed to environmental stresses, they are optically opaque, not easily prepared, and they have limitations to encapsulate, deliver, and protect certain substances. Additionally, surfactant-based delivery systems are optically transparent and thermodynamically stable, but their relatively low loading capacity, and taste are considered as drawbacks. Therefore, biopolymer-based delivery systems are mostly preferred because the fat and surfactant level is reduced [239].

#### 3.2.1. Biopolymer-Based Delivery Systems 

Biopolymers are biodegradable, biocompatible, and biofunctional materials with a structural backbone made of carbon, oxygen, and nitrogen atoms. Biodegradation enables them to break into carbon dioxide, water, organic macromolecular material, biomass, etc. They are divided into four major categories such as: extracted from microorganisms, biomass, petrochemical, and biotechnological products. Polysaccharides (starches, celluloses, alginates, pectins, gums, and chitosan), proteins of animal origin (whey, collagen, and gelatin), proteins of vegetal origin (zein, soya, and wheat gluten), and lipids (bees wax, carnauba wax, and free fatty acids), are biopolymers from biomass products. Most of the work has been conducted based on polysaccharides because of their better properties compared to proteins or lipids [240,241]. Polysaccharides are non-toxic, stable, biocompatible, cheap, hydrophilic, and chemically modified because of their reactive sites. The most used polysaccharides incorporate carbohydrates derived from animals (such as chitosan, and chondroitin sulfate), carbohydrates derived from plants (such as starch, pectin, and guar gum), and carbohydrates derived from other sources (such as alginate derived from algae) [241]. For this review, we discuss dextrins which are synthetic substances obtained from enzymatic degradation of starch and have been considerably used for drug delivery applications [242]. 

##### Starch

**Figure 9:**
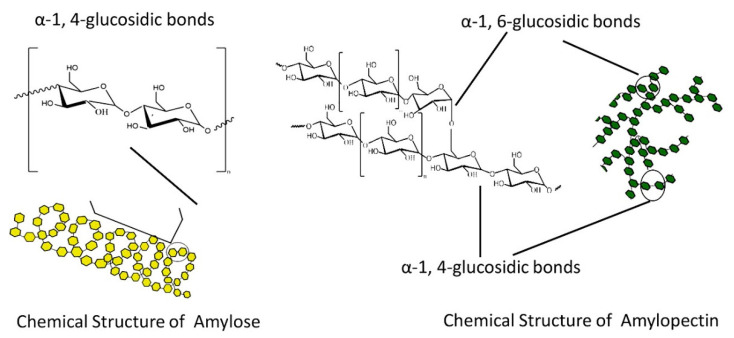
Chemical structure of starch with amylose, and amylopectin.

Starch is one of the most promising natural, biodegradable, abundant, and renewable biopolymers on earth. It is composed of α-amylose (20–30%), and amylopectin (70–80%) (Figure 9). 

α-Amylose is a linear macromolecule of several thousands of glucose residues linked by α-(1→4) bonds in a helix conformation. Amylopectin is a branched molecule with α-(1→6) branch points every 24 to 30 glucose residues on average, and consists mainly of α-(1→4)-linked glucose residues. Amylopectin molecules contain up to 106 glucose residues, making them some of the largest molecules in nature [155,240,242,243]. Starch can be isolated from natural resources such as rice, wheat, corn, and potato. Because of the unique physiochemical and functional characteristics of the starch, various studies suggest that starch-based nano and micro-materials can be utilized for a wide range of applications in pharmaceutical and biomedical research [244,245]. However, the native starch is well-known for the substantial swelling and rapid enzymatic degradation causing the fast drug-release, and limitations in the controlled release of drug delivery systems. To enhance the functionalities and new applications, native starch is modified chemically, physically, genetically, and enzymatically [246]. The modification process alters the physicochemical properties and structural attributes and increases the technological value of the native starch [247]. This has led to the use of starch derivatives that are more resistant to enzymatic degradation, the process of cross-linking, and the formation of co-polymers [248]. Since the mid-1900s, a large-scale starch processing industry has emerged. An excellent review published by Van der Maarel et al. [248] detailed the starch-processing industry. The first step is the liquefaction into soluble and short-chain dextrins, and then the saccharification of the starch-hydrolysate syrup to a high concentration glucose syrup. The mobility of the starch chain is caused by the α-(1→4) glucose units. Due to the various hydroxyl groups starch can be easily functionalized [249]. The starch is chemically or enzymatically processed into diverse products such as starch hydrolysates, starch or maltodextrin derivatives, cyclodextrins, glucose syrups, or fructose [250]. The development of modified dextrin-based chemically cross-linked hydrogels for drug delivery applications is the main focus in this review [16].

##### Dextrin

**Figure 10:**
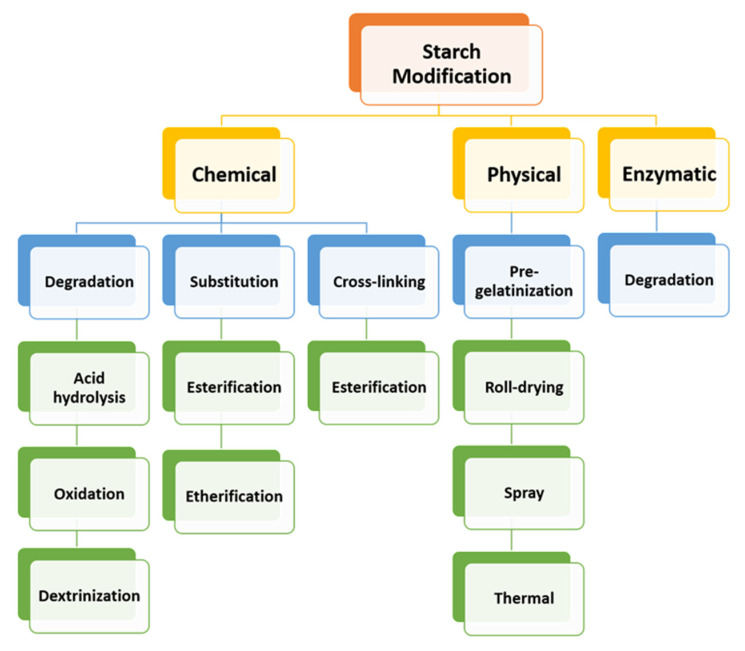
Methods of starch modification.

Dextrin is one of the most noteworthy polymers because of its various features such as hygroscopicity, fermentability, sweetness, stability, gelation, solubility, bioavailability, and molecular compositions. Dextrin is a low-molecular-weight carbohydrate produced by enzymatic and/or acid partial hydrolysis of starch, with the same general formula as starch, but smaller and less complex. It contains α-(1→4) D-glucose units of amylose and the α-(1→4) and α-(1→4,6)-D-glucose units of amylopectin with lower polymerization. During the enzymatic degradation of starch, linear and cyclic dextrins are formed [16,251,252,253,254]. Dextrins are widely used in a variety of applications such as adhesive in the manufacture of textiles, in cosmetics, gummed tapes, and paper, biomedical, and pharmaceutical applications [252]. Because dextrins are easily degraded by α-amylase, the chemical modification can tailor the dextrin structure for satisfying a variety of drug delivery objectives [21]. In general, native starches are often modified as a consequence of their unfavorable properties such as high hydrophilicity, and poor solubility, and herein enhance their application. Although there are several methods of starch modification, as described in Figure 10, chemical modification is the most commonly used. The chemical modification means the introduction of functional groups to the molecule of starch giving characteristic properties [247,255,256]. Among dextrins, cyclodextrin (CD) modifications have become a major area of interest for numerous investigations and, therefore they are widely explored as drug delivery systems.

##### Nanoparticle-Cell Interactions In Vitro, and In Vivo

**Figure 11:**
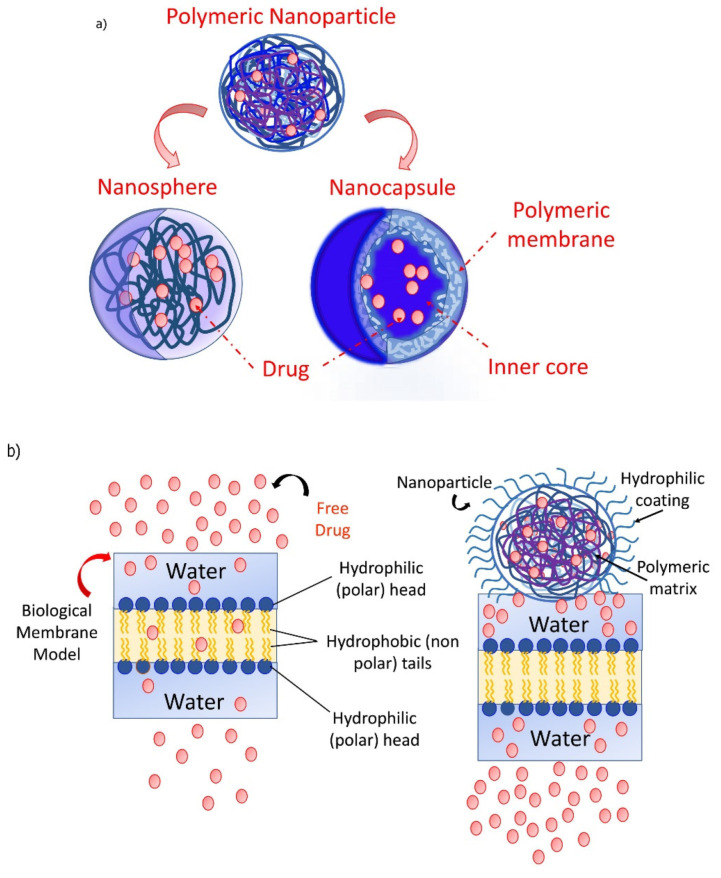
(**a**) Schematic representation of polymeric nanoparticles and (**b**) a model of the interaction between the aqueous phase containing a free hydrophilic drug or drug-loaded nanoparticle and the biological membrane model.

Throughout the years of investigating the interaction of dextrin-based nanomaterials with the cells, researchers stumbled upon questions of how this nanoparticle-based on polymeric materials influences the cellular uptake. With all these nanoparticles at hand, and the new ones, more in vitro and in vivo experiments will be performed after a clear understanding of intracellular trafficking and the fate of the nanoparticles after the endocytotic process [257]. The higher cell affinity and easier uptake of the nanoparticles are due to interactions of the ligands on their surface with the receptors on the cell membranes [258]. The in vitro and in vivo stability are altered by nanoparticle size, stability, responsivity, composition, and surface charge. As already discussed, the therapeutics can be encapsulated within the nanoparticle core, can be chemically conjugated to the polymer, bound to the surface of nanoparticles, or can be entrapped in the polymer matrix. Nanocapsules or cavities surrounded by a polymeric membrane, nanospheres, or solid matrix systems, are the most common forms of polymeric nanoparticles (Figure 11a). 

The nanoparticles encounter diverse interactions with the surface of the cell membrane. Cationic nanoparticles can damage the cell membrane and cause cytotoxicity if are too positively charged, whereas anionic nanoparticles, due to repulsive forces, can fight to contact the cell surface. Therefore, the first contact between a cell and a nanoparticle, dependent on nanoparticle and cell properties, can determine the prospect of the nanoparticle, and its therapeutic potential [259]. A huge nanoparticle with hydrophobic moieties is attached to the membrane because the chemical barrier is one of the fundamental elements that regulate the interaction of nanoparticles with the cell membrane. The strength of the chemical barriers is influenced by membrane compositions. When there are enthalpic interactions between nanoparticle hydrophobic ligands and the membrane interior, caused by lipids with longer tails, an increase in barrier strength is observed [260]. Figure 11b) presents the use of nanoparticles as drug delivery systems to prevail the barriers of drug penetration into cells. An appropriate nanoparticle coating can encourage the insight of hydrophilic drugs through the biological membrane. The hydrophilic drug or free drug can freely diffuse in the aqueous medium. However, the diffusion of this drug can be limited because it is incapable of interacting with the outer-hydrophilic zone of the membrane without passing through the lipophilic layer of the same membrane. Therefore, the outer hydrophilic shell of the nanoparticle is introduced to ensure the interaction with the hydrophilic layer of the membrane, whereas the internal lipophilic core of the particle interacts with the hydrophobic layer of the membrane. A high permeation of the drug is provided. Although the major demands for drug delivery devices are still pending, the in vitro and in vivo results showed the constant improvement that has been made starting from microtechnology, crossing to nanotechnology, and recently viewing the selective drug delivery [10].

#### 3.2.2. General Features of Cyclodextrins and Cyclodextrin-Based Polymers as Delivery Systems Matrices

Cyclodextrins (CDs) are cyclic oligosaccharides produced via cyclodextrin (CD)-glycosyltransferase from starch, by certain microbes such as Bacillus macerans. CDs contain six (αCD), seven (βCD), eight (γCD), or more (α-1,4)-linked α-D-glucopyranose units. The truncated shape of CDs is because of the chair conformation of glucopyranose units with the hydroxyl groups orientated to the cone exterior. The primary hydroxyl groups of the sugar residues are at the narrow edge of the cone, and the secondary hydroxyl groups are at the wider edge [261]. CDs tend to form inclusion complexes because of their lipophilic interior and hydrophilic exterior. The mechanism of the complexation includes the absence of the covalent bonds and the presence of the driving force releasing enthalpy-rich water molecules from the cavity part. CDs can include molecules of size and polarity compatible with their lipophilic inner cavity [262,263]. The formation of hydrogen bridges, between the polar hosts and the primary hydroxyls, establishes simultaneously polar interactions, whereas hydrophobic hosts will be housed inside the CD cavity because of the hydrophobic Van der Waals type interactions [23,264,265]. The versatility of CDs is wide and promising in the pharmaceutical and nutraceutical industries, so far, 26,895 research articles about CDs have been published in PubMed, by typing the word “cyclodextrins” [266].

Even though the unique structure of CDs has fascinated scientists around the world, native CDs are appropriate only for the molecule recognition of a wide range of substrates. Moreover, they have various limitations such as the inability of including certain hydrophilic compounds or high molecular-weight drugs, low aqueous solubility, and toxicity in case of β-CD when administered intravenously [267,268,269]. Therefore, specific applications require overcoming the aforesaid limitations by chemical modifications of CD structures [26,267].

In CDs, hydroxyl groups can be modified by replacing the hydrogen atom or the hydroxyl group with a variety of substituting groups [270]. CD derivatives and CD-based polymers appear as powerful tool. CD derivatives comprise the randomly methylated β-CD, the hydroxypropyl derivatives of β- and γ-CD, sulfobutylether β-CD, and the branched CDs [271], whereas CD-based polymers, containing two or more covalently linked CD-units, can be water-soluble and moderately swelling or insoluble and strongly swelling [272].

CD-based nanosponges (NSs) can easily be obtained by reacting the nucleophilic hydroxyl group of the selected CD with a suitable cross-linking reagent, containing two electrophilic sites, that convert molecular nanocavities into insoluble three-dimensional, nanoporous structures. Widely-used cross-linkers that influence the behavior of the CD units, are epichlorohydrin for hydrophilic NSs synthesis, and diphenyl carbonate (DPC), pyromellitic dianhydride (PMDA), diisocyanates, carbonyldiimidazole (CDI) for hydrophobic NSs synthesis. With a highly porous nanomeric and insoluble nature, CD-NSs are capable of encapsulating a variety of substances, particularly of increasing the solubility of poorly water-soluble drugs, prolonging their release, and improving their bioavailability and stability. Because of these characteristics and their harmlessness, CD-based NSs are used in certain fields such as chemistry, gene delivery, agriculture, cosmetics, food, biomedicine, biotechnology, biocatalysis, etc. In addition, the main area of investigation so far is the pharmacy, in which CD-NSs have been proposed as drug delivery systems [262,263,273,274,275,276,277,278,279]. Therefore, it is no wonder that the demand and the need for an explosive scientific and technological revolution have increased over the years.

##### Historical Developments of Cyclodextrin-Based Nanosponges as Delivery Systems Matrices 

Developing effective CD-based systems that can improve the properties of the nutraceutical has been attractive for many applications, particularly the field of pharmaceuticals as delivery systems. Delivery systems with which the CDs are associated comprise nanoparticles, liposomes, microspheres, hydrogels, and NSs [280]. From a historical point of view, CD-based polymers, reviewed by Petitjean et al., can be observed that have progressed as the result of the enormous research conducted over the years. Despite this, the investigations of CD-based polymers application in pharmaceuticals, food chemistry, and biomedicine, are not as abundant as on the parent CDs applications [281]. CD-NSs appear as advanced drug carriers and, therefore, can also contribute as nutraceutical carriers. As with all of the evidence in the history of CD-NSs [33], detailed by Krabicovà et al., over the years significant progress has been made on CD-NSs synthesis and applications in several scientific and technological fields. According to their chemical composition and properties, CD-NSs with particular attention to the pharmaceutical field, are divided into four generations, as overviewed by Caldera et al. [280]. As already described, poor water solubility, low bioavailability, low stability, low permeability, low efficacy, are some drug features that affect the applications of drugs [23,263,280,282,283]. Therefore, to distinguish between the generations of CD-NSs in the improvement of the aforementioned limitations, several experimental results are compared. The first generation of CD-NSs remains among the most commonly explored NSs as drug delivery systems [284].

##### The First Generation of Cyclodextrin Nanosponges

**Figure 12:**
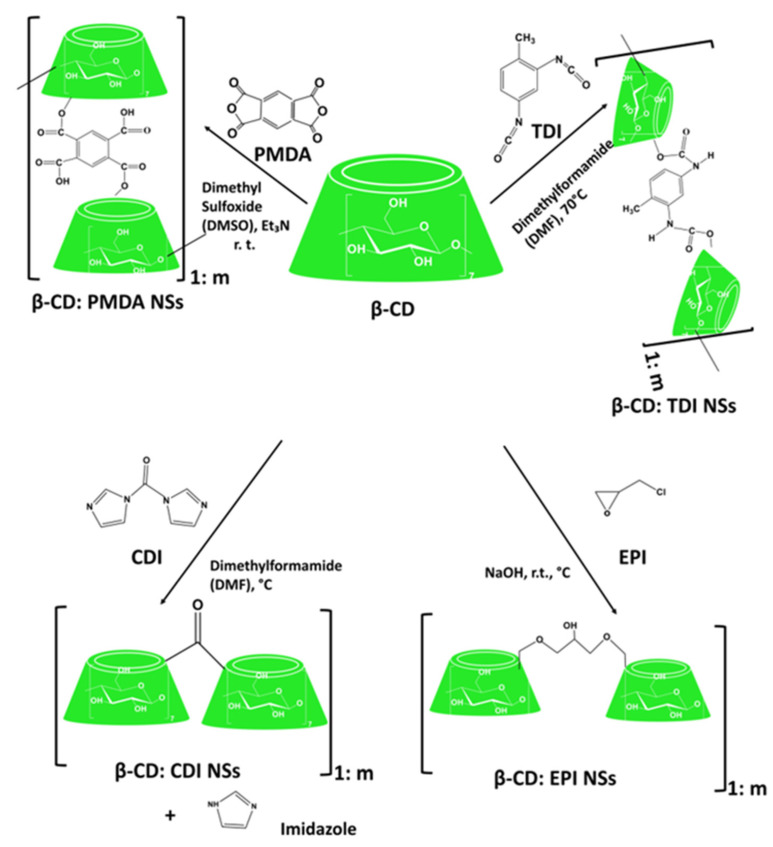
Schematic representation of the synthesis of cyclodextrin (CD)-based ester (PMDA), ether (EPI), carbonate (CDI), and urethane (TDI) nanosponges (NSs).

The first generation comprises urethane (or carbamate), carbonate, ester, and ether CD-NSs [285] (Figure 12).

##### Cyclodextrin-Based Urethane Nanosponges

Diisocyanates such as hexamethylene diisocyanate (HDI), toluene-2,4-diisocyanate (TDI), are used to synthesize urethane (or carbamate) CD-NSs, and are characterized by a very low surface area (1–2 m^2^/g), high resistance to chemical degradation, rigid structure, and a negligible swelling in organic solvent and water [263,285].

A study by Thatiparti and von Recum synthesized CD-based gels by dissolving CD in N, N-dimethylformamide (DMF), and adding 2-isocyanatoethyl 2,6-diisocyanatohexanoate (LTI), and 1,6-diisocyanatohexane (HDI). The antibiotics such as rifampin (RM), novobiocin (NB) sodium salt, and vancomycin (VM) hydrochloride, were loaded to this cross-linked polymer by using a common solvent/solution absorption method. Due to the minimal swelling ability of the synthesized polymer, the percent loading was too low (not more than 3.5%). The antibacterial activity of these antibiotics-loaded gels confirmed a clear zone of inhibition. Additionally, it was observed that the CD-based gels were capable of providing long-term sustained release of these antibiotics. This work developed an anti-infectious drug delivery system [286].

Further, Merritt et al. synthesized CD-based polymers by dissolving γ-CD in N,N dimethylformamide (DMF), or dimethyl sulfoxide (DMSO), and adding hexamethylene diisocyanate (HDI) as a cross-linker. Mitomycin C (MMC), an anti-proliferative drug, was loaded in those polymers. MMC release rates were adjusted to be slower, and more sustained because of the affinity between the MMC and polymer. This therapy, due to less overall exposure to MMC, was suggested to have less clinical risk to the patient and surgical staff compare to traditional treatments [287].

##### Cyclodextrin-Based Carbonate Nanosponges

Active carbonyl compounds such as 1,1′-carbonyldiimidazole (CDI), triphosgene, and diphenyl carbonate (DPC), are used to synthesize carbonate CD-NSs, and are characterized by a low surface area (around 2 m^2^/g), short cross-linking bridges, good stability to acidic and slightly alkaline solutions, and reduced swelling ability [263,285].

Quercetin, a flavonoid with strong antioxidant activity and many others, has pharmaceutically been challenging because it has a very low solubility of 7.563 μg/mL in water. Therefore, Jullian et al. used β-CD and its derivatives such as hydroxypropyl-β-cyclodextrin (HP-βCD), and sulfobutyl ether-β-cyclodextrin (SBE-βCD) to increase quercetin solubility [288]. Furthermore, Anandam et al. investigated the improvement of the chemical stability, and aqueous solubility of quercetin by utilizing the NSs synthesized from β-CD and DPC in five various molar concentrations (1:2, 1:4, 1:6, 1:8, and 1:10). The solubilization efficiency was increased from 7.563 μg/mL (plain quercetin) to 152.543 μg/mL (quercetin-loaded NSs). As the cross-linking ratio influenced the release of quercetin, it was observed from its rapid burst release of 97% within 60 min. These improvements influenced the increased antioxidant activities of quercetin and offered a potential drug delivery system for oral and topical delivery [40].

Curcumin, another nutraceutical with many potential applications, was loaded into βCD-based NSs synthesized using dimethyl carbonate (DMC) by Darandale and Vavia. The in vitro release study of this formulation showed sustained curcumin release. In comparison with plain curcumin (0.4 μg/mL), and the β-CD complex (5.88 μg/mL), it was also observed higher solubilization of curcumin loaded into CD-NSs (20.89 μg/mL) generating a potential drug delivery system for curcumin in cancer treatment [41].

Ansari et al. presented the possibility to administer resveratrol loaded at two different weight ratios of βCD:CDI (1:2 and 1:4) as buccal delivery and topical application. This is because CD-NSs significantly increased the stability, solubility, and permeation of resveratrol, a polyphenolic phytoalexin with many health benefits [40]. Further, Dhakar et al. demonstrated the high encapsulation efficiency of resveratrol (77.3%) and oxyresveratrol (80.33%) into the β-CD:CDI NSs. In comparison to drug molecules alone, solubilization of resveratrol- and oxyresveratrol-loaded NSs was higher, and therefore, a better antioxidant activity was observed [289]. Matencio et al. studied the complexation of the oxyresveratrol with two different weight ratios of β-CD:CDI NSs (1:4 and 1:8) using a new methodology. The apparent inclusion complex constant (KFapp) between β-CD:CDI NSs, and oxyresveratrol was calculated using the UV-Vis measurement and the Benesi-Hildebrand method with modifications. This study represented the feasibility of the drug-CD-based NSs complexation, and the use of oxyresveratrol, which exhibits a wide range of biological activities, as an ingredient in nutraceutical products [290].

Rezaei et al. incorporated thyme essential oil (TEO), a natural phenolic compound with high antimicrobial and antioxidant activity, into β-CD:DPC (1:4, 1:6, 1:8) NSs. The highest amount of loading capacity, encapsulation efficiency, and solubility were of the ratio of β-CD:DPC (1:4) nanosponge. The solubility of TEO increased from 2.7% (free TEO) up to 41% (TEO encapsulated into the nanosponge). A controlled and slow release of TEO from β-CD:DPC NSs was indicated from the in vitro release. Therefore, TEO-loaded into the β-CD:DPC NSs presented the potential to be used as a natural preservative in the food industry [43].

Norfloxacin [291], kynurenic acid [292], tamoxifen [293], rilpivirine [294], acyclovir [295], camptothecin [296], telmisartan [297], babchi oil [298], chrysin [299], paclitaxel [277], sulfamethoxazole [300], ferulic acid [301], melatonin [44], D-limonene [302], azelaic acid [303], paliperidone [304], griseofulvin [305], flutamide [306], econazole nitrate [307], piperine [308], etc., are several other drugs loaded in carbonate CD-NSs. In all these findings it was observed an enhancement of the biocompatibility and aqueous solubility of the aforementioned drugs when they are loaded in NSs compare to CD-inclusion complexes or uncomplexed drugs. The indicated ones present CD-NSs a promising nanocarrier system.

##### Cyclodextrin-Based Ether Nanosponges

Cross-linking agents with epoxide groups such as epichlorohydrin (EPI), bisphenol A diglycidyl ether, ethylene glycol, and diglycidyl ether, are used to synthesize ether CD-NSs, and are characterized by a tunable swelling capability, and high chemical resistance. Although the EPI toxicity, the EPI-based CD-NSs, are widely studied [285].

Machín et al. synthesized CD-EPI insoluble polymer, a novel polymeric hydrogel for the controlled release of anti-inflammatory drugs such as naproxen (NAP), and nabumetone (NAB), and antifungal drugs such as naftifine (NF), and terbinafine (TB). According to this study, these hydrogel matrices were considered as potentially suitable sustained release systems [309].

Rodriguez-Tenreiro et al. synthesized new biocompatible hydroxypropyl-β-CD-based hydrogels using ethylene glycol diglycidylether (EGDE) as a cross-linker and incorporated other structurally related polymers such as hydroxypropylmethylcellulose (HPMC). Diclofenac, a nonsteroidal anti-inflammatory drug [310], was chosen as a suitable candidate to be loaded into the synthesized hydrogels. The hydrogels were able to load and sustain the release of diclofenac for several hours [311].

Gami et al. synthesized novel hydrogels from xylan and β-CD using ethylene glycol diglycidyl ether, as a crosslinker, in an alkaline medium. Two anticancer drugs loaded in hydrogels were curcumin (26%), and 5-fluorouracil (98%). The highest cumulative release of 56% 5-fluorouracil, and 37% curcumin, from the synthesized gels, was observed after 24 h. This study synthesized, characterized, and explored hydrogels as an in-vitro drug delivery vehicle [312].

##### Cyclodextrin-Based Ester Nanosponges

Dianhydrides or di/polycarboxylic acids such as pyromellitic dianhydride (PMDA), ethylenediamine-tetraacetic dianhydride (EDTA dianhydride), butane tetracarboxylic dianhydride, citric acid (CA), etc., are used to synthesize ester- based CD-NSs. These NSs are characterized by a huge absorption of water forming, thus, the hydrogels [263]. 

Appleton et al. proposed β-CD:PMDA NSs as a solution for the oral delivery of insulin. The loading capacity of insulin was 14.41%, and the encapsulation efficiency was 91.40%. In this study it was hypothesized that the β-CD:PMDA nanosponge can be suitable for loading insulin, protecting it from degradation in the stomach, and promoting its intestinal absorption due to controlled release properties [313]. 

Another study made by Argenziano et al., used β-CD:PMDA for the topical delivery of imiquimod (IMQ, 1-[2-methylpropyl]-1H-imidazo[4,5-c] quinoline-4-amine), an immune response modifier. The loading capacity of the IMQ into β-CD:PMDA nanosponge was 14.2%, and the encapsulation efficiency was 96.5%. In this work, a nanomedicine-based topical formulation for the prolonged and controlled release kinetics of IMQ through the skin was developed [314].

Ester nanosponge based on PMDA was also used to maximize the therapeutic efficacy of acetyl salicylic acid (a non-steroidal anti-inflammatory and antipyretic drug) [315], doxorubicin (an anti-cancer drug) [316], meloxicam (a non-steroidal anti-inflammatory drug) [317], rilpivirine (an antiviral drug) [318]. In all instances, a controlled and prolonged drug release, a remarkable increase in the drug solubility and bioavailability, was observed.

##### Other Generations of Cyclodextrin Nanosponges

Other generations of CD-NSs comprise the functionalized CD-NSs, stimuli-sensitive NSs, and molecularly imprinted polymers (MIPs) [285].

**Figure 13:**
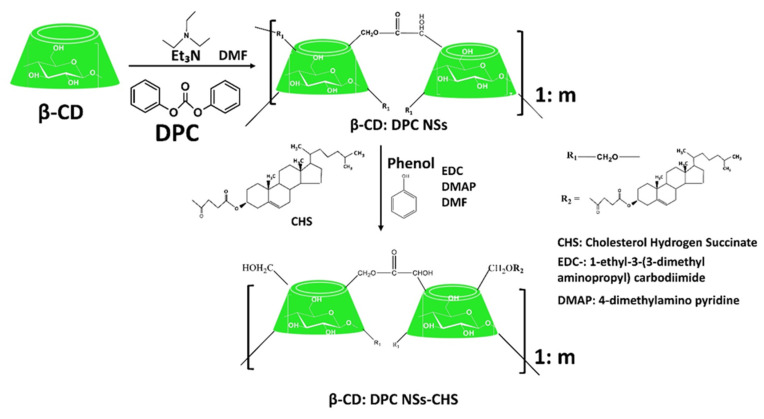
The cholesterol hydrogen succinate (CHS) grafting, in CD-based carbonate NSs (β-CD:DPC NSs), using coupling reaction.

Singh et al. enhanced the cellular binding efficiency of β-CD nanosponge by functionalizing its surface with cholesterol as an endogenous physiological molecule. The cholesterol grafting enhanced the adsorption of the anticancer drug Dox because of the hydrophobic charge on the surface. Therefore, the cholesterol-modified β-CD nanosponge system was proposed to be a site-specific drug delivery carrier improving the solubility and bioavailability of small drug molecules with low water-solubility [319], (Figure 13).

Another study made by Asela et al. developed a new nanomaterial based on βCD based NSs for the transport of phenylethylamine (PhEA), as an antidepressant, and 2-amino-4-(4-chlorophenyl)-thiazole (AT), as an anti-microbial, and anti-inflammatory agent. These complexes were functionalized with gold nanoparticles (AuNPs). In comparison to the native βCD, the loading capacity of βCD NSs was eight times higher for PhEA (90%), and AT (150%). Additionally, βCDNS presented a higher degree of solubilization and complexation efficiency of PhEA and AT than native βCD. The immobilization percentage of AuNPs reached 85%. This study demonstrated the versatile materials (βCD NSs, PhEA, AT, and AuNPs) with an efficient loading capacity for potential applications in the transport of therapeutic agents [320].

**Figure 14:**
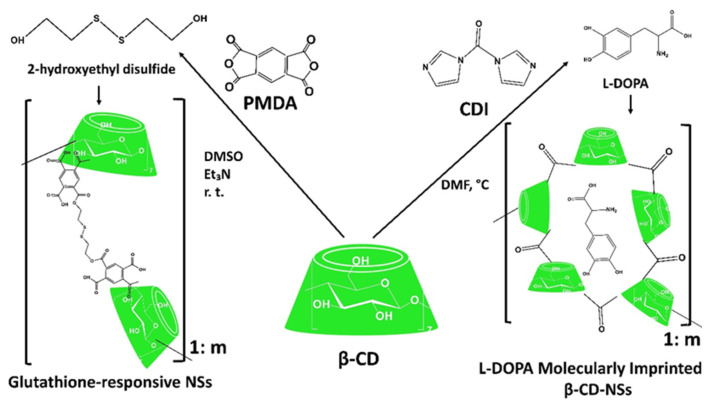
Schematic representation of the synthesis of glutathione-responsive NSs, and molecularly imprinted CD-based carbonate NSs (L-DOPA used as a template).

Although CD-NSs are capable of high drug loading, their non-selectivity remains a challenging task regarding pharmacologic efficacy. To offer the advantage of site-specific drug delivery, stimuli-responsive drug delivery was designed by Palminteri et al. In their study, they presented the synthesis of glutathione responsive (GSH) CD-NSs from PMDA and 2-hydroxylethyldisulfide for improving the tumor-specific delivery of resveratrol. GSH-NSs enhanced the aqueous solubility of resveratrol more than four-fold (201 μg/mL) compared to free resveratrol (46 μg/mL). The encapsulation efficiency was 80.64%, whereas the drug loading was 16.12%, representing the GSH-responsive NSs as an effective delivery system for targeting cancer cells. Daga et al. synthesized the GSH-responsive based NSs using β-CD, 2-hydroxyethyl disulfide, and PMDA as cross-linking agents (Figure 14). Doxorubicin, an anticancer drug, was loaded into GSH-NSs. Dox-GSH-NSs showed a good safety profile, and their hepatotoxicity, observed both in vitro and ex vivo, resulted to be comparable with free Dox. It was observed a slowed and prolonged drug release, and no initial burst effect. No cytotoxicity in vitro and ex vivo was caused by GSH-NSs. Therefore, GSH-NSs are considered a suitable carrier of chemotherapeutic drugs [321].

Coviello et al. presented a study of the use of carbonate CD-NSs, based on DPC as a cross-linker, for targeted drug delivery. Into the synthesized NSs, a novel multi-effective heterocyclic compound, 2-(3,4-dimethoxyphenyl)-3-phenyl-4H-pyrido (DB103), was loaded. DB103, as the ideal drug candidate for drug-eluting stents (DES), was efficiently loaded, and slowly released. The novel DB103-NSs system represented an innovative and credible prototype of formulation used for the local therapy of the vessel’s wall subjected to percutaneous intervention [322].

Deshmukh, Tanwar et al. functionalized the surface-active CDI cross-linked β-CD NSs by lysozyme. Calcium carbonate, and carboxymethyl cellulose, were added to effectively elevate loading efficiency. Lysozyme was delivered in a conformationally stable structure by damaging bacterial cell walls. This was made by catalyzing the hydrolysis of 1,4-β-linkages between N-acetyl-D-glucosamine and N-acetylmuramic acid residues, present in peptidoglycan layer surrounding the bacterial cell membrane, for controlling the calcium release in hypocalcemia condition for 24 h. The NSs formulation is an encouraging carrier for antibacterial protein [323].

Deshmukh et al. synthesized molecularly imprinted polymer (MIP) of PMDA cross-linked βCD-based NSs. The work was compared with non-molecularly imprinted polymer (NIP) NSs, and the glucose was used as a template. The synthesized MIP-NSs showed more significant specificity, and binding for glucose compared to their NIP-NSs [324].

Trotta et al. synthesized a MIP NSs of CDI cross-linked β-CD-based NSs. The L-DOPA, as a non-proteinogenic amino acid widely used for the treatment of Parkinson’s disease (PD), was used as a template. This study showed that the polycarbonate β-CD-based MIP-NSs, as an oral formulation, can be a promising new drug delivery system for the prolonged release, and the protection of L-DOPA [325] (Figure 14).

The safety issue has invoked important remarks regarding the clinical applications of CD-NSs, as drug delivery systems, as they were predominantly synthesized in organic solvents which may cause cellular damage. To overcome the toxic effects, several surveys comprised the green syntheses of a series of CD-based NSs in natural deep eutectic solvents (NADES) [326], and dextrin-based solvent-free NSs synthesis [327], which need to be further studied for medical, and pharma applications. Another promising platform for therapeutic applications that can be considered is a combination therapy with CD-NSs [328].

#### 3.2.3. General Features of Maltodextrins and Recent Trends in Their Applications as Delivery Systems Matrices

Maltodextrin is defined as a hydrolyzed starch product [329] consisting of D-glucose units linked primarily by α (1,4)-glycosidic bonds [330]. Maltodextrins are classified by their values of dextrose equivalent (DE). The DE ranges up to 20 and expresses the number of reducing sugars present in the polymer. Maltodextrins with different dextrose equivalent (DE) values have different properties. A low fraction of glucose and a high fraction of polysaccharides refers to a low DE, and vice versa [25,331,332]. The different reaction sites in repeating glucose moieties of maltodextrins provide wide alternatives in the chemical conjugation process [333]. The characteristics and the physicochemical, nutritional, functional, and technological properties enable the numerous applications of maltodextrins in the food, medical food products, beverage, and the pharmaceutical industry in tablet and powder applications [334]. Maltodextrin is considered the most used starch hydrolysate by the food industry [335].

Sun et al. developed casein-maltodextrin Maillard conjugates to entrap proanthocyanidins (PAs). PAs are a group of polyphenolic compounds with potent antioxidant capacity. However, the practical application as antioxidants is limited because they are easily affected by environmental stress during the processes and storage, and chemically degraded in the gastrointestinal tract. The in vitro release test presented the exhibition of strong protective effects, by casein-maltodextrin-PAs nanoparticles, during the storage and thermal treatments, and PAs bioaccessibility improvement. Thus, the casein-maltodextrin-PAs nanoparticles have been considered as novel antioxidants for applications in pharmaceutical and nutraceutical products. The release rate of encapsulated PAs and free PAs was 30% and 80% after 120 min [336]. The maltodextrins, through the nanoencapsulation, improved the stability, efficiency, and thermal resistance of ellagitannin extracts from pomegranate peels [337].

Further, Gurturk et al. modified liposomes with maltodextrins, and loaded levodopa as the most effective drug to ameliorate the symptoms of Parkinson disease. Therefore, this study suggested an effective way of targeting the blood-brain barrier (BBB) with controlled and sustained drug release properties, improved cellular binding, and lowered cellular cytotoxicity [338].

Lai et al. used maltodextrins as a film-forming material, and glycerin as a plasticizer to enhance the quercetin oral bioavailability. Maltodextrin films provided long-term storage stability, and fast dissolution rate [339].

Helal et al. chemically conjugated the maltodextrin via the formation of the ester bond with vitamin E succinate. The designed bio-conjugates showed higher aqueous solubility, a slighter toxicological effect on the main body organs, and a higher total antioxidant capacity than the vitamin E succinate [333].

Laurent et al. impregnated the ciprofloxacin (CFX) into an artificial polypropylene (PP) abdominal wall implant that was functionalized with citric acid and hydroxypropyl-ϒ-cyclodextrin (HPϒCD), or with maltodextrin. The use of the HPϒCD, and maltodextrins, as two carbohydrate oligomers produced from starch, offered a safe and green process of functionalization, avoiding the common extreme technologies of functionalization such as plasma or radiative technologies. It was observed a higher CFX loading and release over a sustained period in the case of HPϒCD with regard to maltodextrins. The HPϒCD-finished meshes showed antimicrobial activity, and this not only because of the ionic and hydrogen bonding between CFX and CD-finished textile, but also because of the inclusion in the cavity [18].

Maltodextrins were also used to produce proniosomes formulation with a potential application in the delivery of hydrophobic or amphiphilic drugs [340]. Shruthi et al. prepared the resveratrol-loaded proniosomes using maltodextrin, lactose monohydrate, and pullulan as wall materials. The encapsulated resveratrol presented a more consistent and sustained release, and a higher stability under gastric and intestinal conditions [341].

As CDs, maltodextrins may be chemically modified to improve their physical, and functional characteristics. The maltodextrins are less expensive, and have a higher aqueous solubility than that of comparable cyclic dextrins [25,342]. Compared to modified CDs, modified maltodextrins are less studied in pharmaceutical applications. Therefore, the development of modified CDs, which have been shown for various applications, already discussed in this entry, will fully instruct the linear dextrins to be modified and used for nutraceuticals delivery.

Castro-Cabado et al. synthesized the cross-linked system consisting of maltodextrin with citric acid and tartaric acid [343].

Cecone et al. synthesized the sustainable cationic cross-linked polymers, suitable for eco-friendly scaling-up, based on maize-derived maltodextrin (Glucidex 2^®^) as a building block, 1,4-butane-diol diglycidyl ether as a cross-linker, and 1,4-diazabicyclo[2.2.2]octane, imidazole, triethylamine, and guanidine hydrochloride were as supplements to obtain the amine-mediated epoxy ring-opening reaction [344]. These materials, considered genuinely good eco-friendly alternatives, can be further studied as green adsorbents for environmental, medical and pharmaceutical applications because of their high adsorbing feature.

Ivàn Melèndez-Ortiz et al. synthesized the hydrogels with antimicrobial activity based on the maltodextrin, previously modified with glycidyl methacrylate (GMA), copolymerized with acrylic acid (AAc) or acrylamide (Aam) to endow mechanical properties and chemical functionality. This step enabled to host bioactive nanoparticles such as zinc oxide nanoparticles (ZnO). ZnO-loaded hydrogels showed antimicrobial activity against Staphylococcus aureus and Escherichia coli. This study has been promising for the possible application of the maltodextrin-based hydrogels as wound dressings [345].

Yan et al. presented a novel design of entrapping simvastatin (SIM), a hypolipidemic drug, to promote bone regeneration in an injectable maltodextrin-based micelle/hydrogel composite system. The SIM-loaded aldehyde-modified micelles were anchored to the hydrogel network by Schiff base linkage. It was observed a great enhancement of the mechanical strength of hydrogels, good biocompatibility, slow and sustained release, and osteogenic capability of SIM [346].

Furthermore, Demasi et al. selected Kleptose Linecaps^®^ DE17 (LC), apart from CD, as a highly soluble maltodextrin. The study synthesized the carbonate and ester NSs, by cross-linking α-, β-, and ϒ-CD, and LC with PMDA and CDI. Dextrin-based NSs encapsulated ailanthone (Ail) as a natural compound with anti-tumor, anti-malarial, anti-inflammatory, anti-parasitic, anti-feedant, and herbicidal activities, but with low insistence and immediate degradation in organic substrates. The encapsulation process increased and extended the phytotoxic activity, preserved the efficacy, and prolonged the release kinetics of Ail [347].

A safe and sustainable synthesis of NSs based on β-CD, and LC with citric acid in water, using sodium hypophosphite monohydrate as catalyst, was performed by Rubin Pedrazzo et al. The synthesized citrate NSs were able to selectively adsorb a significantly higher amount of heavy metals from aqueous solution [348]. Thanks to the helical structure of the amylose chains, maltodextrins can act as complexing agent demonstrating a strategy to integrate drug loaded carriers into hydrogels for drug delivery and tissue engineering applications [347]. We still lack deeper knowledge of the forces involved in the complex formation of maltodextrins with nutraceuticals, and of the pharmaceutical applications.

## 4. Concluding Remarks

Healthier living, preventive care, and secondary source medication are the focus of the global population since the healthcare costs have risen. The adverse effect of drugs and the risk of toxicity caused a worldwide revolution in nutraceuticals. Nutraceuticals are considered a powerful instrument to facilitate lifespan, optimal health, and quality of life. They act against various nutritionally induced diseases such as obesity, cancers, diabetes, neurodegenerative diseases, cardiovascular disease, allergy, respiratory disorders, arthritis, etc.

In this review, we listed many nutraceuticals classified based on food bioavailability, chemical nature, and mechanism of action, with various health benefits, and summarized common problems that prevent their applications. Similar to the case of any other drug, the health benefits of the nutraceuticals are limited by the low bioavailability, low efficacy, low aqueous solubility, degradation and metabolism, and epithelial permeability.

The polymer-based nanocarriers, with a tripartite structure: monomer, linker, and therapeutic agent, are extensively studied to overcome the aforementioned shortcomings of nutraceuticals. This review shows the use of dextrin-based chemically cross-linked polymer as the most preferred, advanced, biocompatible, and natural system to deliver nutraceuticals. One of the most commonly studied systems is cyclodextrin (CD)-based nanosponges (NSs). CD-NSs are synthesized by reacting CDs with cross-linkers such as carbonyl-diimidazole (CDI), diphenyl carbonate (DPC), pyromellitic anhydride (PMDA), etc. Due to their highly porous nanomeric nature, CD-NSs are found in different applications ranging from chemistry, pharmacy, biomedicine, gene delivery, food and biotechnology, environment, etc.

Nutraceuticals such as curcumin, resveratrol, thyme essential oil, Babchi oil, mitomycin C (MMC), diclofenac, imiquimod, doxorubicin, rilpivirine, melatonin, L-DOPA, etc., are successfully formulated using CD-NSs. CD-NSs are efficient encapsulating agents of delivering nutraceuticals with controlled kinetics through the topical, oral, and parenteral routes. The nanomaterials based on CD-NSs, in contrast to free nutraceuticals and native dextrin units, improved the solubility, biocompatibility, bioavailability, encapsulation, and release capacity of the nutraceuticals, and therefore are considered the most effective delivery systems.

Until now, these nanoparticles have been explored in terms of their physicochemical properties, drug-loading ability, in vitro and in vivo tests. The concerns, such as the specific interaction of the nanoparticles with human organs, tissues, cells, or biomolecules, their effect on human metabolism, and their application in drug delivery, etc., await further intense research, which will be a focus henceforward.

As reviewed above, numerous nanoparticle drug delivery systems, as the most advanced approach contributing to every walk of life, have been prepared. Although maltodextrins are known for a higher aqueous solubility and are less expensive than CDs, their modified forms need to be further studied in pharmaceutical applications. Therefore, the extensive literature about the synthesis, characterization, and applications of the modified CDs, will fully instruct the modified dextrins.

It can be forecasted that the more advanced nanoparticle drug delivery systems will be released and overtake the market.

## Figures and Tables

**Table 1:** The mechanism of the quenching of singlet oxygen by β-carotene.

^1^O_2_	+	β-carotene	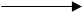	O_2_	+	β-carotene *
β-carotene *			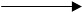	β-carotene	+	energy (heat)
β-carotene *			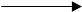	all-*trans*-β-carotene		

β-carotene * -triplet excited carotene.

**Table 2:** Vitamin E reaction with oxygen free radicals.

Vitamin E	+	RO	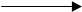	Vitamin E	+	ROH

**Table 3:** The reaction of vitamin E radical with ascorbic acid.

2 Vitamin E	+	ascorbate	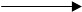	2 Vitamin E	+	dehydroascorbate
